# The Unequal Impact of the COVID-19 Pandemic on Infant Health

**DOI:** 10.1215/00703370-10311128

**Published:** 2022-12-01

**Authors:** Florencia Torche, Jenna Nobles

**Affiliations:** Department of Sociology, Stanford University, Stanford, CA, USA; Department of Sociology, University of Wisconsin–Madison, Madison, WI, USA

**Keywords:** Infant health, COVID-19, Preterm birth, Socioeconomic inequality, Infectious diseases

## Abstract

The COVID-19 pandemic has taken a large toll on population health and well-being. We examine the consequences of prenatal exposure for infant health, through which the pandemic may have lasting intergenerational effects. We examine multiple pathways by which the pandemic shaped birth outcomes and socioeconomic disparities in these consequences. Analysis of more than 3.5 million birth records in California with universal information on COVID infection among persons giving birth at the time of delivery reveals deep inequalities in infection by education, race/ethnicity, and place-based socioeconomic disadvantage. COVID infection during pregnancy, in turn, predicts a large increase in the probability of preterm birth, by approximately one third. At the population level, a surprising reduction in preterm births during the first months of the pandemic was followed by an increase in preterm births during the surge in COVID infections in the winter of 2021. Whereas the early-pandemic reduction in preterm births benefited primarily highly educated mothers, the increase in pre­ term births during the winter infection surge was entirely concentrated among mothers with low levels of schooling. The COVID-19 pandemic is expected to exacerbate U.S. inequality in multiple ways. Our findings highlight a particularly enduring pathway: the long-term legacy of prenatal exposure to an unequal pandemic environment.

## Introduction

The COVID-19 pandemic has had an unprecedented impact on population health and well-being in the United States, where almost 100 million cases of the SARS-CoV-2 virus that causes COVID disease have been recorded by October 2022 and 1.09 million deaths have been attributed to the disease. The impact on health and well-being has not been equally distributed. Cases, hospitalizations, and deaths have been higher among socioeconomically disadvantaged populations (Hawkins et al. 2020; Karmakar et al. 2021) and racial/ethnic minorities—particularly Hispanics, American Indians, and African Americans (Centers for Disease Control and Prevention (CDC) 2021).

A less immediately visible consequence of the pandemic is its potential impact on the next generation: the cohorts exposed to COVID during gestation. The link between *in utero* exposure to COVID and infant health could have direct implications for inequality and the intergenerational transmission of disparities (Hayward and Gorman 2004). An unequal impact of the pandemic would exacerbate profound socioeconomic and racial/ethnic gaps in birth outcomes in the United States (Rauscher and Rangel 2020) and would contribute to already poor infant health relative to that of other wealthy countries (Chen et al. 2016).

Environmental exposures during the prenatal period have a lasting impact on health, cognition, education, and other determinants of economic well-being. Pioneered by the fetal origins approach (Barker 1990, 1995; Barker and Osmond 1986) and elaborated by theories of early human capital formation (Cunha and Heckman 2007; Heckman 2006), research suggests that the prenatal period is among the critical developmental stages of the life course. Even mild shocks can have enduring effects (Almond and Currie 2011). Some of these effects are immediate and translate into infant mortality and health difficulties (Conley et al. 2003) and adversity in childhood (Chatterji, Kim, and Lahiri 2014; Chatterji, Lahiri, and Kim 2014), whereas others are latent and emerge in adulthood (Barker 1990, 1995; Chen and Zhang 2011). However, the impact of prenatal exposures is not deterministic, and later-life interventions and environments can mitigate the impact of early-life insults (Almond and Mazumder 2013). Because such interventions are more likely to be accessible to advantaged groups, compensatory processes could exacerbate intergenerational inequality (e.g., Bernardi 2014; Torche 2018).

Our analysis focuses on preterm birth as a measure of infant health for several reasons. First, preterm birth is the leading proximate determinant of low birth weight and the main predictor of infant morbidity and mortality in the United States (Behrman and Stith-Butler 2007; Lau et al. 2013). Among surviving infants, preterm birth increases the risk of neurodevelopmental delay, chronic lung disease, and other health issues. It also shapes long-term outcomes, including educational attainment and earnings (Luu et al. 2017; Moster et al. 2008; Townley Flores et al. 2021).

Second, although the etiology of preterm birth is not fully understood, it is increasingly recognized as a syndrome with multiple determinants correlated with maternal sociodemographic and behavioral factors, as well as medical conditions and environmental exposures during pregnancy (Behrman and Stith-Butler 2007). The established proximate biological pathways leading to preterm birth include neuroendocrine mechanisms triggered by stress exposure (Baibazarova et al. 2013; Dunkel Schetter 2011); infection (Whidbey et al. 2015); amniotic inflammation (Combs et al. 2014); and pregnancy conditions, such as gestational diabetes and preeclampsia (Hedderson 2003). Given that the determinants of preterm birth are mostly found in conditions and exposures that occur during rather than before gestation (Goldenberg et al. 2008), we expect preterm birth to be sensitive to shocks experienced during pregnancy, such as COVID.

Third, preterm birth is common, affecting more than one in every 10 U.S. infants—one of the highest rates among wealthy nations (Bronstein et al. 2018). Fourth, pre-term birth is sensitive to infectious disease exposure during pregnancy. Indeed, most viral epidemics in the recent past have had important implications for the health of cohorts exposed *in utero*, particularly for preterm birth. In some cases, pregnancy supports the risk of vertical transmission of infection to the fetus. In others, maternal immune responses to infection or infection expression in the placenta can hinder fetal growth or contribute to early delivery. For example, in the context of the recent Zika epidemic in the Americas, maternal infection contributed to Brazilian birth cohorts with an increased prevalence of preterm birth, low birth weight, microcephaly, and stillbirth (Cooper et al. 2019; Rangel et al. 2020). Since the mid-1980s, intrauterine exposure to HIV has contributed to increases in preterm birth globally, especially in sub-Saharan Africa (Wedi et al. 2016).

The potentially uneven consequences of the COVID shock on preterm birth are still largely an open empirical question. Research examining population-level outcomes early in the pandemic documented an unexpected decline in preterm birth in many wealthy countries (Been et al. 2020; Chmielewska et al. 2021; De Curtis et al. 2020; Dench et al. 2022; Fieber 2020; Hedermann et al. 2021; Matheson et al. 2021; Meyer et al. 2021; Philip et al. 2020). As compellingly stated in a *New York Times* article, “During the coronavirus lockdowns, some doctors wondered: Where are the preemies?” (Preston 2020). Researchers have speculated that this beneficial effect might have emerged from positive externalities of the pandemic, including lower exposure to pollution or to other infections, as well as increased time to rest or exercise among pregnant persons (Been et al. 2020; Philip et al. 2020). Other studies have similarly reported an early-pandemic decline in the proportion of U.S. infants born preterm in some areas and nationally (Berghella et al. 2020; Dench et al. 2022; Gemmill et al. 2022; Harvey et al. 2021), but this finding was not universal or persistent over time. Further, some studies found little change in preterm birth in settings as diverse as Massachusetts, Philadelphia, and California (Handley et al. 2021; Main et al. 2021; Wood et al. 2021). Although the evidence is not conclusive because of the small size of many samples and the diversity of methodological approaches and periods considered, one potential explanation for the divergence in findings is the heterogeneity in the pandemic’s effects on pregnancy health.

This study addresses the unequal impact of the COVID pandemic on preterm birth using population-level data for California with universal information about maternal COVID infection. We combine an analysis of individual-level determinants and consequences of maternal COVID infection with a population-level analysis of trends in preterm birth from January 2014 to November 2021. We consider different pathways of influence linking the COVID shock with birth outcomes—including maternal infection, changes in the composition of births, and changes in labor and delivery practices—and the socioeconomic heterogeneity in the impact of pandemic exposure.

### The COVID Shock and Preterm Birth: Pathways of Influence and Socioeconomic Stratification

The CDC began SARS-CoV-2 surveillance in the United States in early January 2020, declaring the developing epidemic a health emergency by the end of the month. By March 2020, state governments around the country began implementing mitigation efforts. California was one of the first states to respond to the COVID pandemic with a statewide shelter-in-place order on March 19, 2020. Although COVID emergency efforts were initially focused on high mortality risk for older adults, early evidence on COVID risk during pregnancy had emerged by fall 2020, suggesting that severe maternal infection may carry health risks for mothers and infants (Pierce-Williams et al. 2020).

Exposure to the COVID pandemic likely shapes preterm birth through multiple mechanisms. First and most obviously, maternal infection during pregnancy could increase the risk of early delivery. Although vertical transmission to the fetus is rare, SARS-CoV-2 infection during pregnancy appears to induce placental abnormalities and may increase the risk of maternal conditions such as preeclampsia, all of which reduce gestation length (Golden and Simmons 2020; Kotlar et al. 2021). Indeed, COVID infection has been shown to increase the probability of preterm birth in several U.S. populations (Allotey et al. 2020; Chamseddine et al. 2020; Conde-Agudelo and Romero 2021; Jafari et al. 2021), especially when mothers experience severe compared with mild symptoms (Lassi et al. 2021; Vouga et al. 2021; Wei et al. 2021).

The direct impact of maternal infection is only one mechanism by which a pandemic may affect population health and health inequality and, depending on the context, might affect only a small portion of pregnant persons. Another set of pathways, then, operates through macro-level exposures. The pandemic shock altered most dimensions of everyday life among pregnant persons, their families, and communities. Economic decline and disruption, policies to curb the spread of infection and support economic well-being, and changes in hospital practices and procedures altered the contexts in which pregnancies took place. These factors, in turn, may have affected well-established proximate determinants of preterm birth, including maternal stress (Dunkel Schetter 2011; Goldenberg et al. 2008; Torche 2011); prenatal care access and quality (Bhatt and Beck-Sagué 2018; Corman et al. 2019); immunity and infection (Goldenberg et al. 2000; Moffett et al. 2015); and parental behavioral responses, such as using prenatal care or smoking (Margerison-Zilko 2014; Strully et al. 2010). Additionally, changes in obstetric and delivery practices and procedures might have increased medically indicated preterm delivery (Villar et al. 2021).

Third, large-scale health and economic shocks might alter the number and composition of persons giving birth through selective fetal loss, migration, or fertility adjustments. The experience of living through a pandemic may cause some groups to revise their fertility timing or face severe restrictions on contraception or abortion access (Lindberg et al. 2021; Marteleto et al. 2020; Rangel et al. 2020). If the COVID pandemic altered *who* gives birth, then any observed changes in infant health might be an artifact of the changing composition of live births. Beach et al. (2022), for example, demonstrated that the reduced welfare of the 1919 U.S. birth cohort originally attributed to intrauterine exposure to the 1918 flu pandemic was instead driven by changes to the composition of people giving birth in the pandemic’s aftermath. Any analysis of pandemic health effects must account for these potential compositional changes.

### Socioeconomic Disparities in the Impact of the COVID Shock on Infant Health

The exposure to COVID and its corollaries is embedded within social structures that reflect established stratification patterns in American society. Evaluating the stratified impact of COVID in this context requires considering the kind of exposure COVID is: (1) a novel health threat that (2) may be at least partially prevented by behavioral and local­level structural modifications to reduce risk, which (3) causes broader disruption to family arrangements and economic well-being and (4) poses disparate risks based on cumulated health disadvantage. We rely on two theoretical approaches—fundamental cause theory and cumulative disadvantage approaches—to formulate testable hypotheses linking this type of exposure to unequal outcomes.

Fundamental cause theory suggests that socioeconomic inequality in health persists over time because socioeconomic advantage comprises multiple resources, such as money, prestige, power, and social connections (Clouston and Link 2021; Freese and Lutfey 2011; Link and Phelan 1995; Phelan et al. 2004). These resources can be mobilized to avoid disease risk and minimize consequences once disease occurs, no matter which mechanism is more relevant at any specific time.

The fundamental cause approach predicts that socioeconomic position will be more salient when the factors affecting health are, as in the case of COVID, preventable and therefore allow for resources to be deployed (Clouston and Link 2021; Phelan et al. 2004). Even in the absence of inequalities in infection risk or severity, disparities in the impact of infection could emerge from differences in health care access and treatment. As the medical sociology literature has documented, advantaged populations are more likely to benefit from health­enhancing innovations (Chang and Lauderdale 2009) and to receive more attention from health care providers (Hernandez 2013; Lutfey and Freese 2005; van Ryn and Burke 2000). These factors might result in different standards of care among pregnant persons from different socioeconomic backgrounds.

Observed stratification patterns of COVID infection for the general population are consistent with fundamental cause theory. Socioeconomically disadvantaged and racially minoritized persons were more likely to be exposed to COVID-related risk factors: they were more likely to live in crowded conditions, to hold jobs that could not be performed remotely, and to be concentrated in public-facing occupations, all of which make maintaining social distancing difficult to afford (Garcia et al. 2021; van Dorn et al. 2020; Webb Hooper et al. 2020). Risk-reduction strategies, such as working from home and wearing masks, were more likely to be adopted by socio-economically advantaged groups (Papageorge et al. 2021; Wright et al. 2020). As vaccines became available in the spring of 2021, stratification of uptake was also evident. Lower vaccination rates among African Americans, Hispanics, those with lower education, and rural residents (Rawal et al. 2022) were partly shaped by differences in access and by differences in exposure to disinformation, misinformation, racism, and abuse in medicine that, in turn, fostered distrust.

Not surprisingly, given the stratification of risk factors, research suggests socio­economic disparities in COVID infection among pregnant populations. Pregnant persons with low educational attainment, racial/ethnic minorities, and those living in disadvantaged neighborhoods have been found to be more likely to be infected or have COVID symptoms during pregnancy (Ames et al. 2021; Goldfarb et al. 2020; Joseph et al. 2020; Karasek et al. 2021; Prasannan et al. 2021; Sakowicz et al. 2020).

Indirect pathways linking the COVID pandemic to infant health emerging from economic and family disruption also suggest marked socioeconomic stratification. Early in the pandemic, risk factors such as employment loss, food insecurity, and heightened anxiety were much more likely to disrupt the lives of individuals in vulnerable communities, including those with low levels of education and racial/ethnic minorities (Adams-Prassl et al. 2020; Perry et al. 2021; Perzow et al. 2021). In contrast, potentially beneficial corollaries of the pandemic—such as the reduction in work- and commute-related strain, air pollution, and exposure to other infections during pregnancy—likely benefited advantaged populations that were able to transition to remote work without substantial cost (Clouston et al. 2021).

Supplementing fundamental cause theory, the cumulative disadvantage approach suggests that disparities in exposure to infection may be compounded by disparate vulnerability to its consequences for maternal and infant health. Diverse strands of this approach, including allostatic load (McEwen and Stellar 1993) and weathering (Geronimus 1992; Geronimus et al. 2006), suggest that the deleterious effects of long-term economic hardship and social exclusion and discrimination over the life course may be a predisposing factor for the influence of a novel insult. For example, minoritized persons or persons living in poverty might be more vulnerable to infection because of health deterioration and comorbidities from lifetime adversity (Lu and Halfon 2003). Indeed, these populations experience an unequal burden of comorbidities, including overweight, hypertension, diabetes, and depression, which might make them more vulnerable to the impact of COVID infection (Lorch et al. 2012; Nagahawatte and Goldenberg 2008). Departing from predictions suggested by the cumulative disadvantage approach, however, more recent evidence suggests limited socioeconomic variation in the severity of disease given COVID infection among pregnant persons (Galang et al. 2021; Ko et al. 2021; Metz et al. 2021), even if substantial stratification has been found for the general population (Ebinger et al. 2021; Wiemers et al. 2020).

The fundamental cause and cumulative disadvantage approaches are theoretically distinct. However, they offer similar predictions about the unequal impact of the COVID pandemic on infant health: the same groups of people who faced disproportionate exposure to pandemic risks and fewer resources to manage those risks also began the pandemic with a larger burden of health risks accumulated over their lives.

On the basis of these theoretical approaches, we formulate the following hypotheses:

#### Hypothesis 1:

Socioeconomically disadvantaged persons are more likely to be infected with COVID during pregnancy because of a higher prevalence of risk factors and reduced ability to reduce risk.

#### Hypothesis 2:

Disadvantaged pregnant persons with a COVID infection during pregnancy are more likely to experience preterm birth because of a heavier burden of comorbidities and differential treatment by the health care system.

#### Hypothesis 3a:

The population­level impact of COVID on infant health will be stratified by socioeconomic advantage as a result of both direct (infection among pregnant persons) and indirect (economic and family disruptions and policy responses to the pandemic) mechanisms.

#### Hypothesis 3b:

The population­level impact of the pandemic might even vary in direction across groups defined by socioeconomic advantage, with direct and indirect pathways potentially increasing preterm births among disadvantaged groups and the positive externalities of the pandemic potentially benefiting advantaged groups and reducing preterm births.

### Data and Analytic Plan

#### Data

We rely on detailed birth records for all infants born in California between January 2014 and November 2021. We obtained early-release restricted-access natality microdata based on birth certificates from the California Department of Public Health. The natality data set contains information about the date of birth, infant characteristics (e.g., sex, gestational age), and mother’s characteristics (e.g., education, race/ethnicity, ZIP code of residence). The total sample size is 3,667,903. To the natality data, we merged sociodemographic information from the ZIP code tabulation area of the pregnant person’s residence obtained from the American Community Survey 2015–2019.

#### Measuring COVID Infection

Starting in June 2020, California birth certificates recorded both confirmed and presumptive cases of COVID infection among all persons giving birth. Confirmed cases are indicated by tests verified by the CDC laboratory, whereas presumptive cases result from tests conducted by a state or local laboratory but not yet confirmed by the CDC at the time the birth certificate was recorded. Following CDC guidelines, both presumptive and confirmed cases are considered a diagnosis of COVID.^1^

Although testing could plausibly have occurred at any point during pregnancy, recording protocols at the hospital level indicate that COVID diagnoses are virtually always based on screening upon hospital admission. As a result, the birth records largely capture variation in infection at the time of delivery hospitalization. From the perspective of infant health, this is an important point in the pregnancy at which maternal COVID infection operates (Glynn et al. 2021; Narang et al. 2020). It is also the only moment in pregnancy when complete population information about COVID infection is possible in any setting. Nearly all analyses of COVID infection’s population health effects are thwarted by missing information on infected people who are not tested. To address this issue, we contacted the majority of the more than 420 hospitals and birthing units in California^2^ and ascertained whether universal testing on admission was administered and if so when it started for 75% of births occurring since June 2020, when birth certificates started including this information. Of these births, 72% occurred in a facility with confirmed universal testing. We use this data set to test the robustness of results by limiting the sample to births that occurred in a facility with confirmed universal testing (see Additional Analyses and Robustness Checks section). The absence of universal maternal infection data earlier in the pregnancy has implications for the interpretation of our findings—because of this feature of the data, it is possible that our results provide a lower bound on the burden of COVID infection on preterm birth. We discuss this issue in the final section.

### Analytic Plan

We combine analyses at the individual and population levels to examine the implications of the pandemic on infant health. We first consider individual­level determinants of COVID infection during pregnancy and the consequences of maternal COVID infection on preterm birth. Then, we scale up to the population level and examine changes in preterm birth since the onset of the COVID pandemic for all births; we also stratify the sample by maternal educational attainment as an indicator of socio-economic advantage.

#### Individual-Level Determinants and Consequences of Maternal COVID Infection

To characterize differences in COVID infection risk and the consequences of maternal infection on preterm birth across social groups, we estimate fixed­effects linear probability models predicting, respectively, the probability of maternal SARS-CoV-2 infection during labor and delivery hospitalization and the probability of preterm birth (i.e., <37 completed weeks of gestation based on the obstetric estimate of gestation at delivery). The analysis considers the period when COVID infection status among persons giving birth was first recorded on California birth certificates (June 22, 2020) to the most recent date available at this writing (November 28, 2021). We restrict the observations to singletons to limit the influence of other determinants of preterm birth that accompany multiple-birth pregnancies. Fixed effects for birth facility account for differences in COVID testing protocols, labor and delivery protocols, and other institutional characteristics that might shape preterm birth. Fixed effects for calendar week account for trends in COVID infection and temporal changes in testing shared across facilities.

The sociodemographic predictors included in the models are mother’s race/ ethnicity (Hispanic, Black, White, Asian, and other race/ethnicity), educational attainment (less than high school, high school diploma, some college, bachelor’s degree, and graduate degree), and socioeconomic disadvantage of the ZIP code of mother’s residence. We construct this latter measure with information on the proportion of adult residents in the ZIP code with a high school diploma or less, median household income, the proportion Hispanic, the proportion Black, the Gini index, the proportion foreign-born, the proportion of households below the poverty line, and the proportion of individuals without health insurance. We used principal component analysis to calculate the ZIP code disadvantage index, extracting the first component and dividing it into quartiles.

Prior scholarship suggests that socioeconomic gradients in maternal and infant health vary across racial/ethnic groups (Acevedo-Garcia et al. 2005; Green and Hamilton 2019). To examine potential socioeconomic stratification among minoritized populations, we test for interactions between race/ethnicity and educational attainment. All models adjust for age (19 or younger, 20–24, 25–29, 30–34, 35–39, and 40 or older), parity (1, 2, and 3 or more), and foreign-born status. All models use robust standard errors clustered at the birthing facility level.

#### Population-Level Analysis

To assess population-level trends in preterm birth during COVID, we aggregate individual-level data into a weekly time series of preterm birth rates from January 1, 2014, to November 28, 2021. We create time-series data sets for all singleton births and across levels of mother’s educational attainment. Time-series data contain sources of temporal autocorrelation, including trend, seasonality, and the tendency for high and low values to persist over several periods, which can confound the effect of exposure (Catalano et al. 2008). We estimate autoregressive integrated moving average (ARIMA) models, which allow disturbances to follow a linear autoregressive moving average specification accounting for sources of temporal autocorrelation. ARIMA estimation uses prepandemic time-series data (January 2014–February 2020) to predict counterfactual preterm birth rates expected during the pandemic (March 2020–November 2021). We evaluate differences between observed and predicted postpandemic preterm birth rates to capture changes in preterm birth attributable to the pandemic environment. ARIMA model selection was based on visual inspection of correlation and autocorrelation plots (Pankratz 1983) and fit statistics, including the Akaike information criterion (AIC) and the Bayesian information criterion (BIC).^3^ The preferred ARIMA specification for the entire population and education­specific groups is a seasonal multiplicative model with one first­difference term, one moving average term, and one seasonal 52-week difference parameter capturing annual seasonality (i.e., ARIMA[0,1,1] × [0,1,0,52]).

## Results

### The Unequal Risk of COVID Infection During Pregnancy

**Fig. 1:**
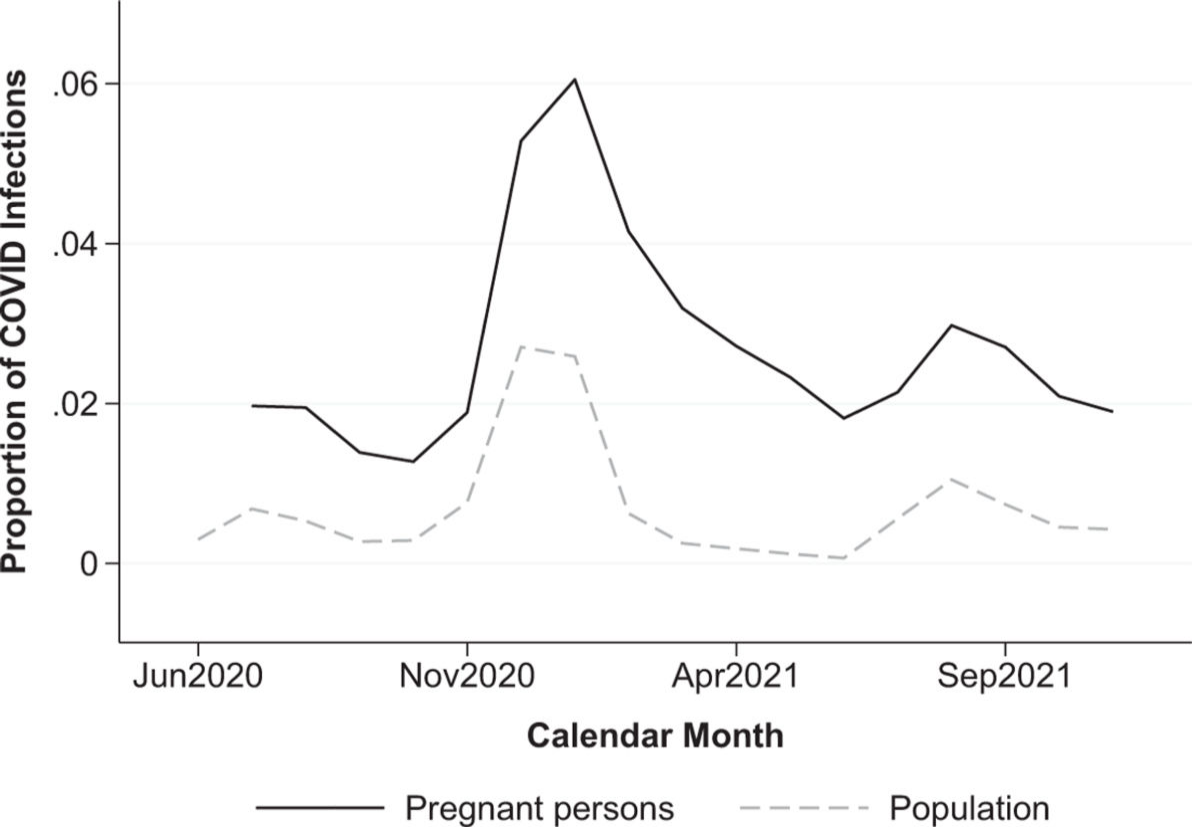
Trends in COVID infection among persons giving birth and the general population, June 2020–November 2021. *Sources:* Data for pregnant persons are from the California Department of Public Health; data for the general population are from *The New York Times* COVID data repository (https://github.com/nytimes/covid-19-data ).

COVID cases in California exhibited a gradual increase through the summer of 2020, a reduction in the fall, and a large spike in the winter of 2021 (Figure 1). After a sharp reduction in cases in the spring of 2021, a comparatively minor rise in cases was observed in the fall of 2021 (our data do not yet document the Omicron-related infection surge). Figure 1 also displays infection trends among persons giving birth and for the entire California population. The substantial overlap in the two time series supports the claim that maternal COVID infection was measured at the time of delivery for most birthing persons.

**Fig. 2:**
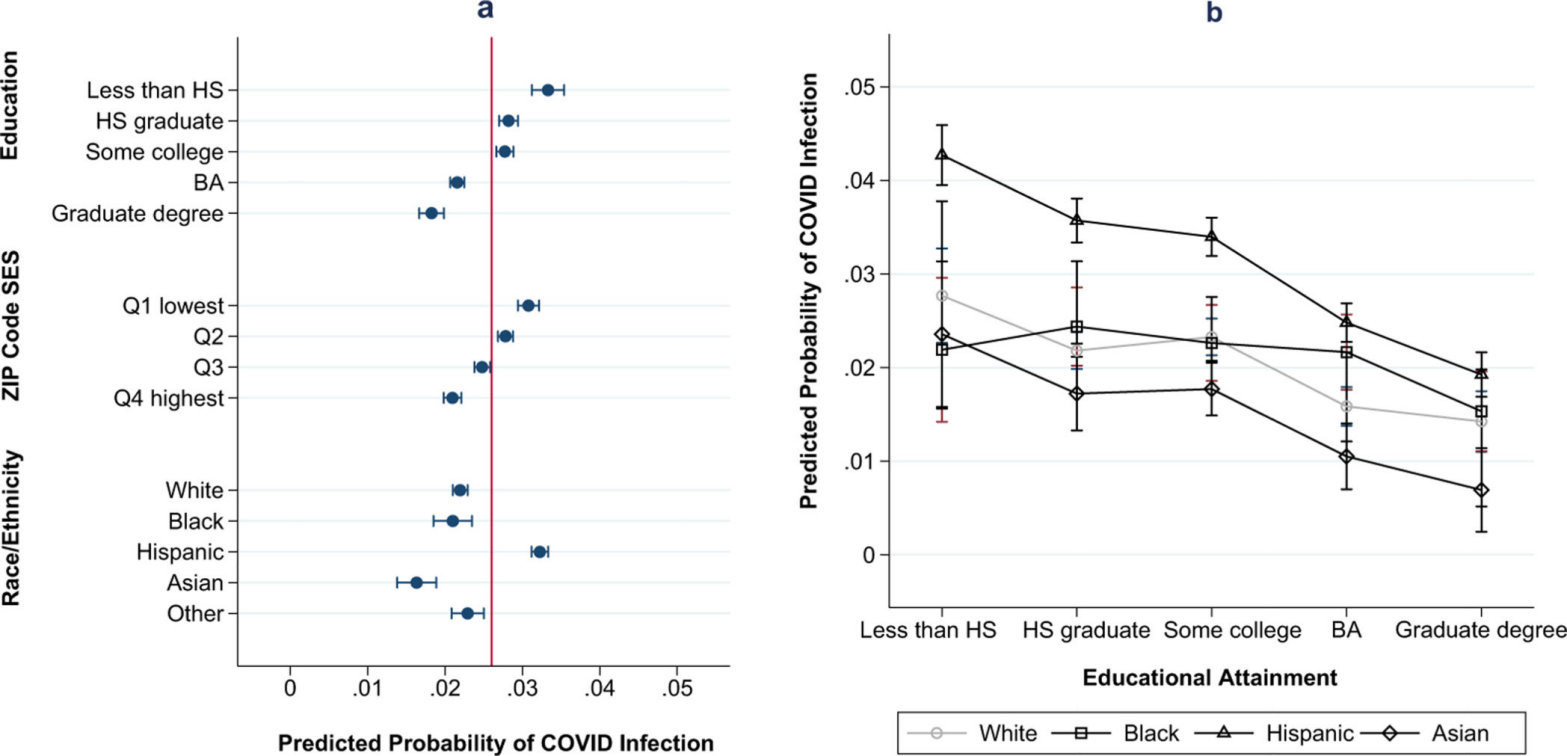
Sociodemographic predictors of COVID infection at the time of delivery among persons with a singleton birth in California from June 22, 2020, to November 28, 2021. Predicted probabilities of COVID infection at the time of labor and delivery were obtained from linear probability models with fixed effects for hospital/place of birth and week of birth. Solid and open symbols indicate parameter estimates, and the bars depict 95% confidence intervals. SARS-CoV-2 infection was measured at the time of delivery hospitalization. In panel a, additional predictors include mother’s age, age squared, birth parity, foreign-born status, and ZIP code of residence socioeconomic quartile. The vertical red line indicates the overall infection rate. In panel b, the statistical specification includes cross-product terms between the mother’s race/ethnicity indicator variables and the mother’s educational attainment. HS = high school. BA = bachelor’s degree. SES = socioeconomic status. *Source:* California vital statistics.

Between June 22, 2020, and November 28, 2021, 2.6% of pregnant persons tested positive for SARS­CoV­2 infection. Infection risk was highly stratified by socioeconomic resources. Our fixed­effects analysis shows a marked gradient by educational attainment and ZIP code socioeconomic disadvantage (Figure 2, panel a). The probability of COVID infection reached 3.3% among mothers with less than a high school diploma and dropped monotonically with increases in educational attainment, reaching 1.8% among mothers with a graduate degree. Net of educational attainment, mothers residing in the poorest 25% of ZIP codes had a 3.1% likelihood of COVID infection, compared with only 2.1% for those residing in the wealthiest ZIP codes. With these socioeconomic gradients held constant, Hispanic mothers exhibited the highest risk of infection, at 3.3%; differences across other groups were smaller and statistically insignificant (2.2% for Whites, 2.1% for Blacks, 1.6% for Asians, and 2.3% for other racial/ethnic groups). We observe some differences in infection risk by age and foreign­born status: younger mothers and those born outside the United States were at higher risk of infection. However, these determinants of infection risk pale in comparison with disparities based on maternal educational attainment, ZIP code socioeconomic disadvantage, and Hispanic ethnicity (see parameter estimates and significance tests in Table A1; all tables and figures designated with an “A” are available in the online appendix).

Prior scholarship suggests that educational and other socioeconomic gradients in maternal and infant health may vary across racial/ethnic groups (Acevedo-Garcia et al. 2005; Green and Hamilton 2019). We therefore examine whether these dimensions of disadvantage interact in the configuration of risk. The effect modification analysis finds marked variation in the association between educational attainment and risk of infection across racial/ethnic groups (Figure 2, panel b). Although an educational gradient exists for all groups, the gaps are particularly pronounced for Hispanic mothers. Hispanic women with less than a high school diploma display the highest risk of infection, at approximately 4.4%—almost twice the risk of Hispanic coethnics with a graduate degree and comparable to that of low-education women of other races/ethnicities.

#### The Link Between Maternal COVID Infection and Preterm Birth

**Fig. 3:**
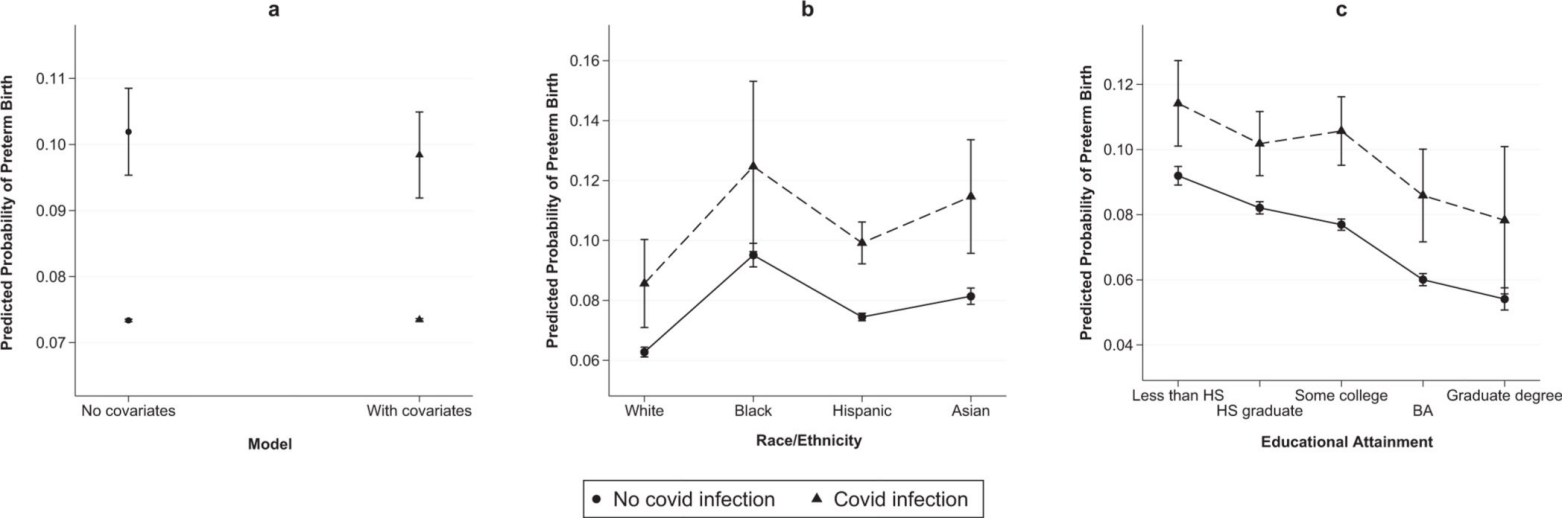
Association between COVID infection at the time of delivery and preterm birth among persons with a singleton birth in California from June 22, 2020, to November 28, 2021. Predicted probabilities of preterm birth were obtained from linear probability models with fixed effects for hospital/place of birth and week of birth. Solid symbols indicate parameter estimates, and vertical bars depict 95% confidence intervals. Results are from a linear probability model with fixed effects for birth facility and week of birth. Covariates include the mother’s age, age squared, birth parity, education, race/ethnicity, foreign-born status, and ZIP code of residence socioeconomic quartile. In panel b, the model specification includes a cross-product term between the mother’s race/ethnicity indicators and an indicator for COVID infection during pregnancy. In panel c, the model specification includes a cross-product term between the mother’s educational attainment and an indicator for COVID infection at the time of delivery. SARS-CoV-2 infection was measured at the time of delivery hospitalization. HS = high school. BA = bachelor’s degree. *Source:* California vital statistics.

Maternal COVID infection at the time of labor and delivery predicts a large increase in the probability of a preterm birth (Figure 3, panel a; refer to Table A2 for parameter estimates and significance tests). These associations are estimated from linear probability models adjusting for the week of birth, facility fixed effects, and the aforementioned covariates. In the model adjusting for mother’s socioeconomic characteristics, COVID infection during pregnancy is associated with a 2.5-percentage-point increase in the probability of preterm birth (from 7.3% to 9.8%)—a 34% increase. This increase is large compared with the impact of environmental stressors or policies intended to support infant health (Hoynes et al. 2015; Torche 2011).

The stratification of COVID infection risk during pregnancy might be compounded by disparities in the association between maternal infection and preterm birth. As the fundamental cause and cumulative disadvantage approaches predict, the impact of infection on infant health might be more acute among disadvantaged populations because of a stronger burden of comorbidities, reduced access to medical technologies, and dissimilar standards of care. We do not find evidence supporting a stronger negative association between COVID infection and preterm births among disadvantaged groups defined by maternal race/ethnicity and educational attainment (panels b and c in Figure 3; Table A2). The link between maternal infection and pre-term birth is similar across all educational and racial/ethnic groups (*p* > .05 for all pairwise comparisons). In sum, our analysis shows that mothers with low levels of education, living in disadvantaged ZIP codes, and who are Hispanic experienced an elevated risk of preterm birth during the COVID pandemic. This excess risk is due to a higher probability of COVID infection rather than to a more detrimental impact of infection on preterm birth.

#### Population-Level Trends in Preterm Birth During the COVID Pandemic

We have documented a substantial and unequal impact of maternal COVID infection on preterm birth. *Ceteris paribus*, such an impact should result in a population-level increase in preterm birth that closely patterns infection trends if maternal infection were the only pathway linking the pandemic to birth outcomes. In such a case, we should observe an increase in the preterm birth rate during the summer of 2020 and, particularly, during the winter of 2021, when COVID infections peaked in California. Indirect behavioral and environmental pathways might, however, offset the direct effect of infection, at least among some subgroups defined by socioeconomic advantage.

**Fig. 4:**
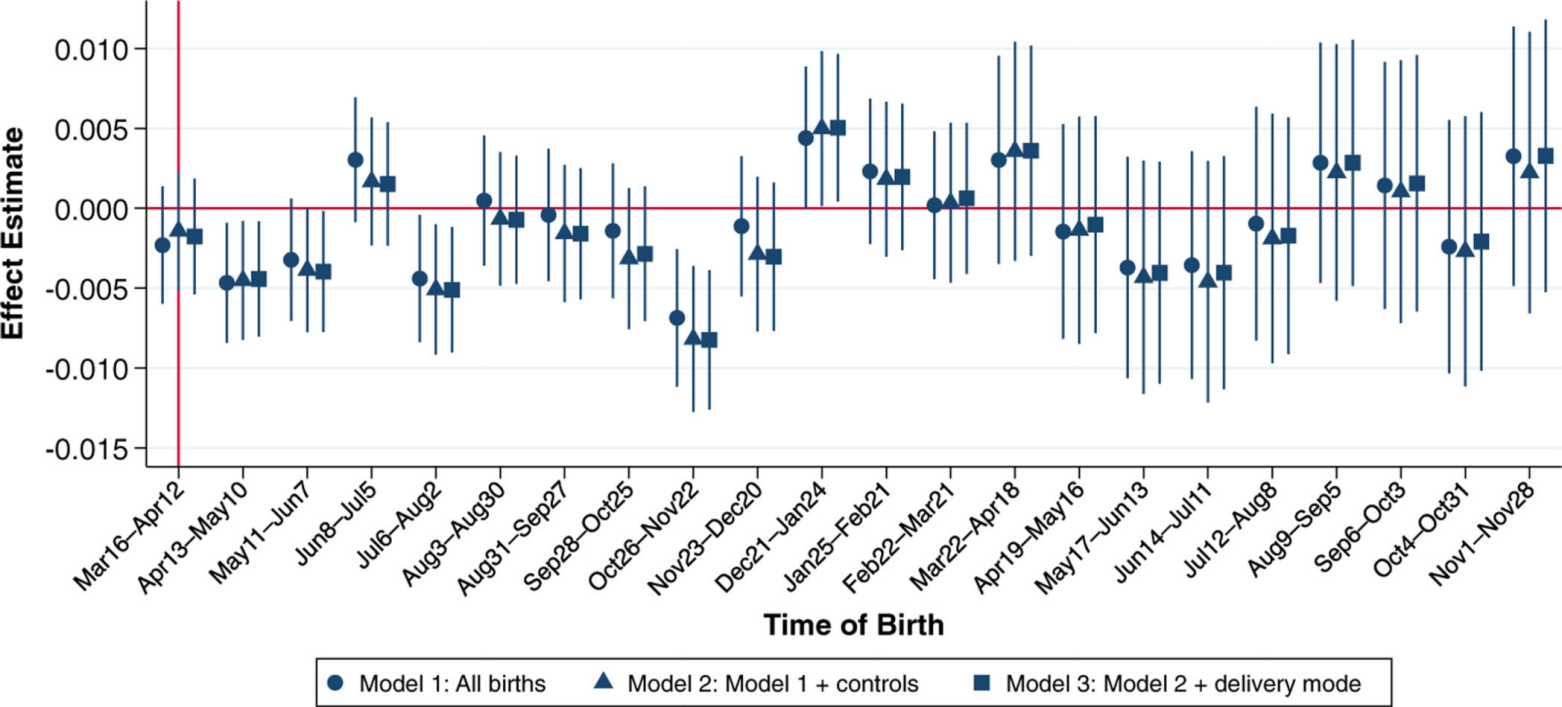
Time-series ARIMA analysis predicting changes in the preterm birth rate after the pandemic onset among all persons giving birth in California from March 2020 to November 2021. Symbols indicate parameter estimates, and vertical lines depict 95% confidence intervals. Parameter estimates represent risk differences between the observed preterm birth rate and the rate expected as a function of autocorrelation from January 2014 to February 2020 (the prepandemic period). Model 2 includes the following covariates: mother’s age, age squared, birth parity, education, race/ethnicity, foreign-born status, and ZIP code of residence socioeconomic quartile. Model 3 adds controls for cesarean section and labor induction.

The population-level ARIMA analysis shows substantial changes in the preterm birth rate during the COVID pandemic compared with counterfactual expectations based on past trends. Consistent with prior findings of a decline in preterm births in some populations, we observe a reduction in preterm births during the first months of the pandemic. This reduction is statistically significant during April, May, July, and November 2020 (Figure 4, Model 1; Table A3). This change is reversed in the winter of 2021, and a significant increase in preterm births is observed in January 2021 as COVID infections surged. The preterm birth rate then returned to levels expected based on prepandemic trends in February 2021 and remained stable thereafter. In terms of magnitude, these changes are substantial. For example, preterm births declined by 0.7 percentage points (from a baseline of 7.2% to 6.5%) in November 2020 and increased by 0.5 percentage points in January 2021. Given the number of births in California during these periods, these figures translate to 195 fewer infants born preterm in November 2020 and 140 more infants born preterm in January 2021 because of the pandemic.

The population-level decline in preterm birth during 2020, when the infection rate was relatively low, is consistent with arguments that policy and behavioral responses intended to reduce COVID risk had beneficial spillover effects on infant health. Alternatively, the composition of births observed after the onset of the pandemic might have been altered through fertility adjustments, fetal loss, or selective migration of the pregnant population, leading to positive selectivity among pregnancies resulting in live births. Fertility adjustments are particularly likely to account for the observed decline in preterm birth in November 2020, when the first cohort of infants conceived in a pandemic environment were born.

To address the impact of compositional change on preterm birth trends, Model 2 in Figure 4 adds covariates to the ARIMA model to account for the sociodemographic makeup of persons giving birth: mother’s age, age squared, birth parity, education, race/ethnicity, foreign-born status, and ZIP code socioeconomic disadvantage. These results yield virtually identical trend estimates to those of Model 1, suggesting that compositional change based on observed sociodemographic attributes does not account for the observed 2020 decline in preterm births. Even if unmeasured maternal characteristics (e.g., risk aversion or fertility desires) could still induce unobserved positive selectivity, these factors would have to be orthogonal to observed covariates, which is unlikely.

An alternative explanation of the changes in preterm birth during the pandemic is changing obstetric practices or criteria for medically indicated preterm delivery to reduce the infection risk of pregnant persons and health care providers. Model 3 in Figure 4 adjusts for the mode of delivery, including controls for cesarean section and labor induction. Postpandemic trends in preterm birth remain unmodified, suggesting that changes in labor and delivery protocols are an unlikely explanation for COVID-era changes in preterm birth.

Our population-level analysis demonstrates that the pandemic had a substantial impact on infant health and that this impact changed over time. We observe a beneficial decline in preterm births during the first few months after COVID was declared a global pandemic, followed by an abrupt increase in the proportion of preterm births as infections peaked in the winter of 2021 and an equally abrupt return to values predicted based on prior trends. In what follows, we examine potential mechanisms as well as socioeconomic disparities in these trends.

**Fig. 5:**
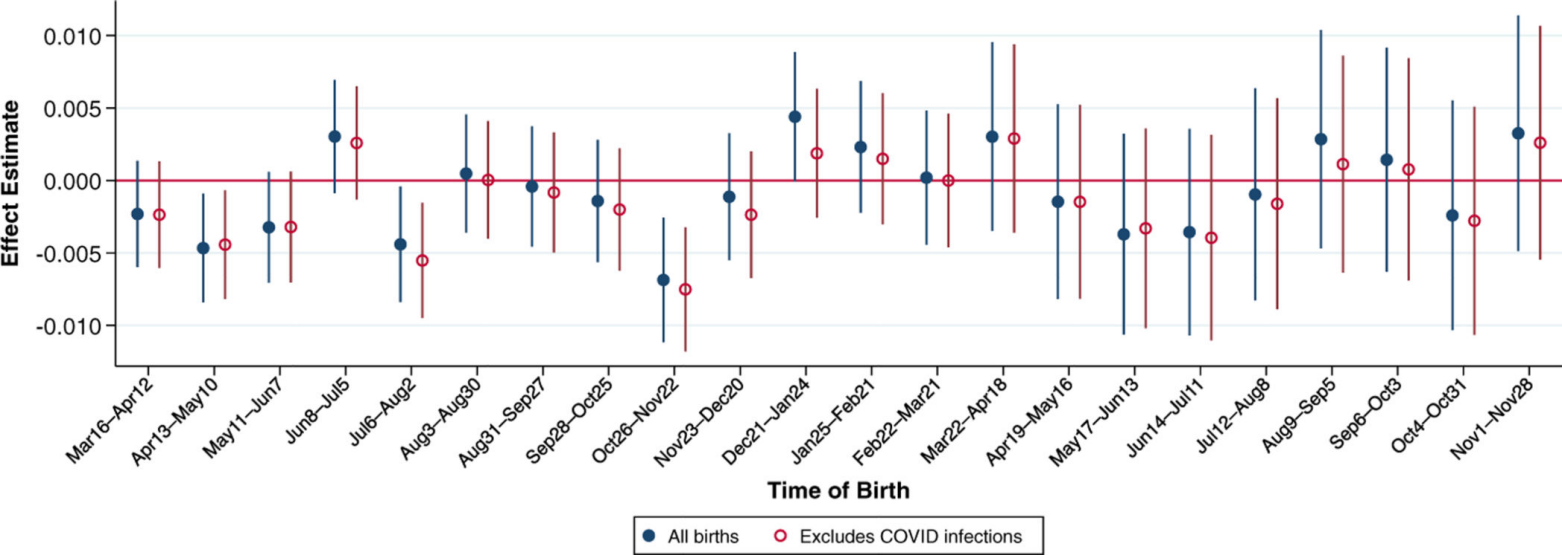
Time-series ARIMA analysis predicting changes in the preterm birth rate after the pandemic onset among all persons giving birth and persons giving birth without a COVID infection at the time of delivery in California from March 2020 to November 2021. Symbols indicate parameter estimates, and vertical lines depict 95% confidence intervals. Parameter estimates represent risk differences between the observed preterm birth rate and the rate expected as a function of autocorrelation from January 2014 to February 2020 (the prepandemic period).

To address the role of maternal COVID infections as a direct pathway to the pre-term birth increase during the surge of infections in the winter of 2021, we evaluate trends that exclude pregnant persons infected with COVID. A comparison of ARIMA estimates suggests that maternal COVID infection accounts for a substantial portion of the preterm birth increase in that period (Figure 5 and Table A3). When we restrict the sample to COVID-negative persons giving birth, the January 2021 increase in preterm births drops from 0.50 to 0.15 percentage points and is no longer statistically distinguishable from predicted values.^4^ This finding suggests that the rise in preterm births in the winter of 2021 was largely driven by maternal COVID infection.

Aggregate preterm birth trends might mask substantial variability by maternal socioeconomic status. As the fundamental cause and cumulative disadvantage approaches would predict, disadvantaged families might have been the most affected by the winter surge of COVID infections because of the direct toll of infection and the disruptions caused by the infection surge. In contrast, the beneficial spillovers of policy and behavioral responses intended to reduce risk early in the pandemic may have been confined to socioeconomically advantaged groups. To address inequalities in the pandemic’s population-level impacts, we stratify the sample by maternal educational attainment, distinguishing mothers with a high school diploma or less, some college, and a bachelor’s degree or more.

**Fig. 6:**
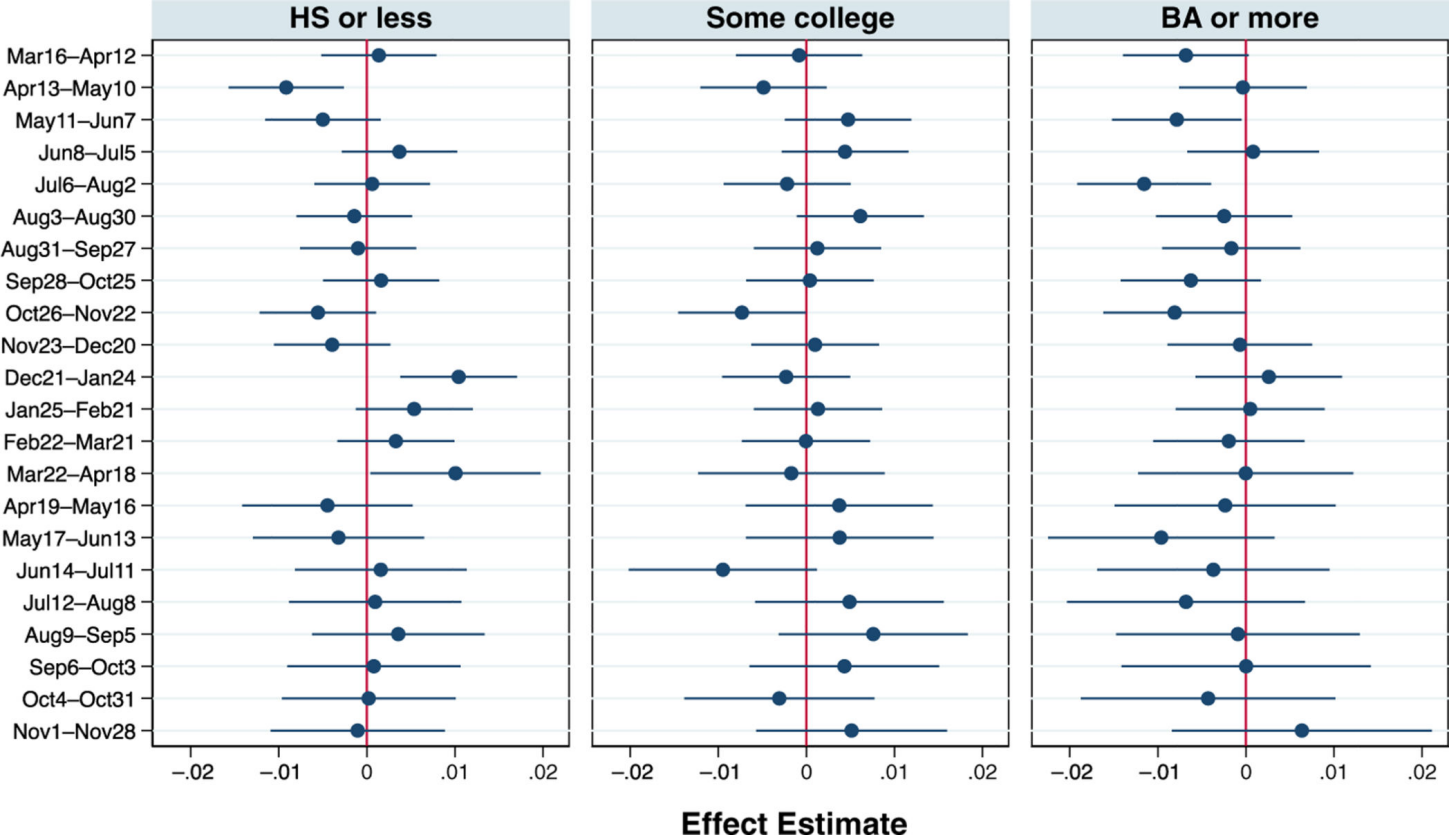
Time-series ARIMA analysis predicting changes in the preterm birth rate after the pandemic onset among subgroups of persons giving birth, by educational attainment, in California from March 2020 to November 2021. Symbols indicate parameter estimates, and vertical lines depict 95% confidence intervals. Parameter estimates represent risk differences between the observed preterm birth rate and the rate expected as a function of autocorrelation from January 2014 to February 2020 (the prepandemic period). HS = high school. BA = bachelor’s degree.

Figure 6 shows a highly stratified overall impact of the pandemic on preterm births: mothers with a high school diploma or less were the only group that experienced an increase in preterm births during the winter 2021 infection surge (parameter estimates are shown in Table A3). The increase was substantial, reaching 1.1 percentage points in January and April 2021. (We also observe substantive increases in preterm birth in February and March for this group, although they are not statistically distinguishable from predicted trends.) Strikingly, preterm birth did not increase during the winter of 2021 or at any point thereafter among pregnant persons with higher levels of schooling, likely reflecting their reduced exposure to COVID infection.

Our analysis also suggests that the beneficial reduction in preterm birth in the early months of the pandemic was greater and more persistent among the most advantaged mothers—those with a bachelor’s degree or more. Mothers with a high school diploma or less experienced a reduction in preterm birth only in April 2020. In contrast, statistically significant declines in preterm birth benefited highly educated mothers in March, May, and July 2020 (note that the difference in parameter estimates across groups is not itself statistically significant at the 5% level, so these findings provide suggestive evidence; see, e.g., Gelman and Stern 2006).

#### Additional Analyses and Robustness Checks

We performed additional analyses to assess the robustness of our findings. We replicated the analyses of COVID infection during pregnancy and the consequences of infection on preterm birth while restricting the sample to facilities with confirmed universal testing (Table A4) and using a binary logit instead of a linear probability specification (Table A5). Both replications yielded estimates substantively identical to those reported in the main analysis. Additionally, we estimated models for the association between maternal COVID infection and preterm birth controlling for a larger set of prepregnancy factors known to predict preterm birth (maternal hypertension, diabetes, large fibroid tumors, asthma, and smoking) and measures of prenatal care (the trimester in which prenatal care started and the number of prenatal care visits). Although validation studies using medical records have shown that these measures have limited accuracy in birth certificates (in contrast to gestational age and sociodemographic information), these models provide a stronger safeguard against confounding and account for the mediating role of prenatal care use. These models yielded results similar to those reported in the main analysis (Table A6), reducing the risk of unobserved confounding on the estimated impact of maternal COVID infection on preterm births.

We implemented several extensions of the population-level analysis of trends. First, we estimated ARIMA models organizing the time-series data by time of conception rather than time of birth (Figure A1). This reformulation evaluates potential discrepancies between cohort and period consequences of the pandemic. Results were largely substantively identical to those reported in the main analysis; the exception was an expected temporal lag in the cohort analysis, given that preterm births occur, by definition, earlier than term births. Second, we used an alternative strategy to remove autocorrelation in time­series data: we extracted trends with a Hodrick–Prescott filter and seasonality with indicator variables for the week of the year and then implemented a Prais–Winsten regression approach to account for temporal auto-correlation (Figure A2). Finally, we conducted a placebo test considering preterm birth trends among births occurring 24 calendar months before the onset of the pandemic, from March 2018 to November 2019 (Figure A3). These additional analyses produced results consistent with our reported findings on trends for the entire population and by subpopulations defined by maternal educational attainment.

## Discussion

Our findings show that the pandemic has had substantial, dynamic, and sharply stratified consequences for preterm birth rates, a relevant indicator of early­life health. Several prior studies have pointed to the initial drop in preterm births across wealthy countries as a potential positive externality of the COVID shock—an unexpected population health benefit attributed to sheltering in place. Our analysis suggests that these findings are incomplete and could be misleading. Akin to findings for other countries, we observe a population-level decline in preterm births during the early stage of the pandemic. However, this decline was short-lived and was followed by an increase in preterm births during the surge of COVID infections in the winter of 2021. Furthermore, when we consider socioeconomic variation in preterm births, it becomes clear that trends in population means conceal important heterogeneity.

As Hypothesis 3 predicted, the population-level reduction in preterm births early in the pandemic was larger and more persistent among socioeconomically advantaged pregnant persons. In contrast, the increase in preterm births during the winter surge of COVID infections was entirely concentrated among pregnant persons with a high school diploma or less, with no observable consequences for those with higher levels of schooling. These findings are entirely consistent with predictions from the fundamental cause approach and highlight the relevance of multiple and flexible resources that advantaged populations can mobilize to reduce infection risk and benefit from positive externalities of early­pandemic policy responses (Clouston and Link 2021; Clouston et al. 2021). Specifically, in terms of infection risk, as Hypothesis 1 predicted, pregnant persons with more schooling and living in advantaged communities were much more likely to evade COVID infection during the first 18 months of the pandemic, likely because they were disparately able to engage in risk-reduction responses, such as working from home, avoiding public transit, testing regularly, and (for those giving birth after the spring of 2021) receiving vaccines when available.

Our analysis also reveals an important instance in which predictions from the fundamental cause and the cumulative disadvantage approaches appear not to have materialized. Departing from Hypothesis 2, we do not find a stronger association between maternal COVID infection and preterm births among disadvantaged mothers relative to their more-resourced peers. The similarity of these associations across social groups was robust to the inclusion of a large set of covariates (Table A6). Despite well-established disparities in maternal comorbidities and standards of care in the United States, a COVID infection was not more detrimental for pregnant persons with low education or for racial/ethnic minorities during the period studied here.

Still, the potential for an unequal impact of maternal COVID infection on pre-term births deserves close monitoring as the pandemic evolves. Hospital case studies suggest that COVID disease severity during pregnancy increases risks for infant health (Lassi et al. 2021; Vouga et al. 2021; Wei et al. 2021). The recent winter 2022 infection peak driven by the Omicron variant (not yet captured in the data used here) occurred when access to vaccination and antiviral therapy may have induced inequality in disease severity and may result in emergent socioeconomic gaps in the impact of COVID infection during pregnancy on preterm births. If so, it would provide further support for the fundamental cause and cumulative disadvantage theories of health stratification in populations: as tools to mitigate disease severity emerge, we might expect infection to have differential impacts on pregnant people that align with their ability to access vaccination and antiviral treatment.

Furthermore, we do not find evidence that preterm births were strongly patterned with economic decline and recovery. Previous research suggests that macroeconomic shocks increase preterm births for infants exposed in the first trimester and sometimes the second trimester (Margerison et al. 2019; Noelke et al. 2019). If the early-pandemic economic contraction had a systematic effect on preterm births, we would have expected to see increases in preterm births in August 2020 through November 2020, corresponding with births occurring four to seven months after the unemployment spike in April 2020. We would also have expected preterm births to be concentrated among people with less schooling, who were disproportionately likely to lose jobs early in the pandemic. We do not see evidence that this pattern materialized in fall 2020.

Overall, our findings underscore the limits of restricting our understanding of pandemic effects to aggregate means and the need to examine socioeconomic inequality. Although aggregate data are often the only evidence available early in emergent health crises, aggregate trends are potentially misleading when considered alone and provide little guidance for targeting scarce resources. Our findings also underscore the relevance of identifying the specific mechanisms driving unequal impacts of health shocks. A key insight of the fundamental cause theory is that socioeconomic gradients in health persist because the specific resources that advantaged populations mobilize change over time as specific risk factors evolve. We find that inequality in infant health early in the COVID pandemic emerged from the unequal ability to benefit from positive externalities of sheltering in place. Later in the pandemic, inequalities emerged from the differential ability to avoid infection risk as cases peaked.

As intuitive as these findings might seem *a posteriori*, there is nothing self-evident about them. We could have found inequality to be driven by an uneven impact of COVID infection during pregnancy on preterm birth or by the uneven toll of the economic disruption during the pandemic. As the pandemic evolves, new sources of disparities will emerge. Because complete vaccination and use of booster vaccinations among pregnant populations appear to be sharply stratified by education and race/ethnicity (Kricorian et al. 2021; Rawal et al. 2022; Troiano and Nardi 2021), vaccine access and acceptability are likely to become a central axis of inequality.

Our findings have concerning implications for the longer term effects of the pandemic. Although our findings address preterm births and cannot be extended to other measures of infant health, preterm birth is a relevant predictor of health and attainment over the life course. Infants born preterm are more likely to incur complications at birth and are in greater need of health and developmental intervention in the first few years. Many of these sequelae have substantial financial costs, and the consequences of preterm birth may even extend into adult health, education, and earnings. Our findings indicate that the pandemic exacerbated burdens on disadvantaged sub-populations that may have fewer resources to manage it and has almost certainly contributed to intergenerational stratification.

Our results also emphasize the importance of ongoing attention to maternal and fetal health in the context of pandemics and health crises more generally. The COVID pandemic has appropriately brought attention to the management of elderly health. However, maternal and child health was disproportionately excluded from the early pandemic response: less than 1% of the National Institutes of Health 2020 COVID funding went to maternal health, and less than 3% went to pediatrics (Balaguru et al. 2022). This exclusion, alongside early evidence of preterm birth reductions, may have resulted in overlooking significant population health costs.

Correctly interpreting the findings from this analysis requires attention to several limitations of our data. A relevant feature of infection data is that testing reflects hospital-administered tests at the time of admittance for delivery and may not capture earlier-pregnancy infection. Research on COVID has been largely silent about the timing of infection as a prognostic factor—partly because no pregnant cohort has yet to be tested continuously over pregnancy. Some evidence and theoretical models about viral mechanisms suggest that early-pregnancy infection might also increase preterm births (Badr et al. 2021; Valdespino-Vázquez et al. 2021). If so, and given that repeated infection during pregnancy is extremely rare, early-pregnancy infection would be a confounder in our analysis: it both reduces the odds of infection at the time of birth and increases the odds of preterm birth. The substantial increase in preterm births during the peak of infections in the winter of 2021 suggests that late-pregnancy infection is critical for infant health, but the strong association we document between maternal COVID infection and preterm birth could be an underestimate of the true burden of COVID infection on infant health if early-pregnancy infection is also harmful. Only a cohort of pregnant persons with repeated testing over the pregnancy will provide adjudicative evidence.

In addition, we describe the impact of the pandemic on infant health in California, a large and socioeconomically diverse state comprising 12% of all U.S. births. We see no reason to expect less inequality in the impact of COVID in other states. In fact, given that California’s policy responses to the pandemic were more comprehensive, durable, and arguably protective of vulnerable populations than those of other states, we might expect that the consequences of the pandemic on infant health are even more stratified by socioeconomic advantage elsewhere. We invite future research to examine this possibility as data become available. One salient finding in the California context warrants additional consideration: we cannot fully explain the much higher infection risk of Hispanics compared with other racial/ethnic groups, even if we account for sociodemographic covariates. One relevant possibility is that the Hispanic population’s excess risk results from heightened occupational exposure to infection. In California, the Hispanic population includes a larger share of people working in settings with COVID outbreaks, such as residential care and food-processing facilities (California Department of Public Health 2021). To address this possibility, we extended our analysis of determinants of COVID infection and preterm birth to include occupational sources of COVID infection risk of both parents, including occupational exposure to infection and proximity to others. Table A7 displays the results based on occupation data for both parents from California birth certificates. We found that such sources of risk shape the probability of maternal infection but only marginally account for the Hispanic population’s excess risk. We suspect that classifications of occupational exposure currently available are limited in their ability to capture exposures among disadvantaged populations with high rates of occupational informality (Andrasfay et al. forthcoming; Pebley et al. 2021). Additional research in California and other settings is needed to better understand the multiple sources of racial/ethnic disparities in risks emerging from COVID beyond conventional markers of socioeconomic advantage.

Finally, we do not attempt to identify the causal effect of social distancing policies on infant health, given that the COVID pandemic involved multiple treatments (i.e., other large-scale exposures) that evolved alongside social distancing policies. Earlier studies attributed postpandemic changes in birth outcomes to policy responses, but such responses cannot be considered exogenous. Evidence indicates that people adjusted their behavior and hospitals altered health care protocols to reduce the risk of COVID infection independent of social distancing policies (Berry et al. 2021; Davis-Floyd et al. 2020; Yan et al. 2021).

The evidence here indicates key avenues for future research. Although the mortality costs of the COVID pandemic are unambiguously clustered among the elderly, pregnant populations—and, by extension, those of reproductive age—warrant serious public health attention. Further, the longer run outcomes for children born during the pandemic, particularly those born into low-resourced families with a higher risk of COVID exposure, require special attention and monitoring over time. Several safety-net programs have been tested during the pandemic, and attention to their inequality-mitigating effects will be important as the cohorts that were prenatally exposed age. Finally, the study underscores that attention to population heterogeneity during large-scale health crises is critical. Although aggregate data provide early surveillance, they likely mask substantial variation, ranging from harmful effects for some groups to neutral or even positive effects for others. As we have shown, early-release administrative data with key information on socioeconomic heterogeneity provide a critical resource for developing strategies to support infant health in the context of health crises.

## Supplementary Material

Appendix

## Footnotes

1 See https://www.cdc.gov/nchs/data/icd/COVID-19-guidelines-final.pdf.

2 More than 99% of California births take place in a hospital or midwifery facility.

3 ARIMA models arguably provide the most flexible approach to adjust for multiple forms of temporal autocorrelation. To test sensitivity, however, we also estimated Prais–Winsten regressions on time-series data purged from trend and seasonality (see Additional Analyses and Robustness Checks section). These regressions produced results similar to those presented here.

4 The difference between the two estimates is not statistically significant (*p* = .40).
