# Supplementary material for: The Unequal Impact of the COVID-19 Pandemic on Infant Health
Source: Demography. Author manuscript; Available in PMC 2023 Jun 16. (PMC10273135; doi:10.1215/00703370-10311128)
Supplement: Appendix [file NIHMS1881514-supplement-Appendix.pdf]

The Unequal Impact of the COVID-19 Pandemic on Infant Health

Florencia Torche and Jenna Nobles

Online Appendix

Index

Table A1. Parameter estimates and significance testing, linear probability fixed effects models predicting person giving birth's COVID infection at the time of delivery. Singleton births in California June 22, 2020 – November 28, 2021.

Table A2. Parameter estimates and significance testing, linear probability fixed effects models predicting preterm birth. Singleton births in California June 22, 2020 - November 28, 2021.

Table A3. Parameter estimates and significance testing, ARIMA models predicting changes in the preterm birth rate after the onset of the COVID pandemic. Singleton births in California January 1, 2014- November 28, 2021.

Table A4. Analyses predicting person giving birth's COVID infection at the time of delivery and preterm birth, restricting sample to birthing facilities with confirmed universal testing. Singleton births in California June 22, 2020 - November 28, 2021.

Table A5. Binary logistic fixed effects models predicting person giving birth's COVID infection at the time of delivery and preterm birth. Singleton births in California June 22, 2020 - November 28, 2021.

Table A6. Analyses predicting person giving birth's COVID infection at the time of delivery and preterm birth including a larger set of covariates. Singleton births in California June 22, 2020 - November 28, 2021.

Table A7. Analyses predicting preterm birth adjusting for occupation-based proximity to others and occupation-based exposure to infection of both parents. Singleton births in California June 22, 2020 – November 28, 2021.

Figure A1. ARIMA analysis predicting changes in the preterm birth rate after the onset of the pandemic comparing models dating births by time of birth and by time of conception. Singleton births in California January 1, 2014- November 28, 2021.

Figure A2 Analysis predicting changes in the preterm birth rate after the onset of the pandemic using Prais-Winsten regression to account for autocorrelation in time-series data. Singleton births in California January 1, 2014- November 28, 2021.

Figure A3. Placebo ARIMA analysis predicting changes in the preterm birth rate two calendar years prior to the onset of the pandemic. Singleton births in California January 1, 2014- November 28, 2019.

Table A1. Linear probability fixed effects models predicting COVID infection at the time of delivery. All singleton births in California June 2020-November 2021.

	Model 1<br>Main effects	Model 2<br>M1+interaction terms
Education: Less than high school (reference)		
Education: High school graduate	-0.005***	-0.006***
	(0.001)	(0.002)
Education: Some college	-0.006***	-0.007***
	(0.001)	(0.002)
Education: BA	-0.012***	-0.015***
	(0.001)	(0.002)
Education: Graduate degree	-0.015***	-0.021***
	(0.001)	(0.002)
Race/ethnicity: Hispanic (omitted)		
Race/ethnicity: White	-0.011***	-0.014***
	(0.001)	(0.003)
Race/ethnicity: Black	-0.012***	-0.022***
	(0.002)	(0.005)
Race/ethnicity: Asian	-0.016***	-0.018***
	(0.002)	(0.004)
Race/ethnicity: Other	-0.010***	-0.013*
	(0.001)	(0.005)
White*HS graduate		0.001
		(0.003)
White*Some college		0.003
		(0.003)
White*BA		0.007*
		(0.003)
White*Graduate degree		0.010**
		(0.003)
Black*HS graduate		-0.005
		(0.005)
Black *Some college		0.000
		(0.000)
Black *BA		0.010*
		(0.004)
Black *Graduate degree		0.009*
		(0.004)
Asian*HS graduate		0.019***
		(0.005)
Asian *Some college		0.018***
		(0.005)
Asian *BA		0.001
		(0.007)
Asian *Graduate degree		0.000
		(0.005)
Other race/ethnicity*HS graduate		0.002
		(0.005)
Other race/ethnicity *Some college		0.004
		(0.005)
Other race/ethnicity *BA		0.007
		(0.005)
Other race/ethnicity *Graduate degree		-0.005
		(0.006)
Foreign-born	0.003***	0.002***
	(0.001)	(0.001)
Age: 19 or younger		
Age: 20-24	-0.002	-0.001
	(0.002)	(0.002)
Age: 25-29	-0.005**	-0.005**
	(0.002)	(0.002)
Age: 30-34	-0.009***	-0.009***
	(0.002)	(0.002)
Age: 35-39	-0.012***	-0.011***
	(0.002)	(0.002)
Age: 40 and older	-0.011***	-0.011***
	(0.002)	(0.002)
ZIP code SES: Wealthiest quartile (reference)		
ZIP code SES: Quartile 2	0.004***	0.004***
	(0.001)	(0.001)
ZIP code SES: Quartile 3	0.007***	0.007***
	(0.001)	(0.001)
ZIP code SES: Poorest quartile	0.010***	0.010***
	(0.001)	(0.001)
Parity: First birth (reference)		
Parity: Second birth	0.002***	0.002***
	(0.000)	(0.000)
Parity: Third birth or higher	0.005***	0.005***
	(0.001)	(0.001)
Constant	-0.023***	-0.026***
	(0.005)	(0.005)
Observations	545231	545231

Cluster robust standard errors in parentheses. + p<.10 * p<.05, ** p<.01, *** p<.001. COVID infection measured at time of labor and delivery hospitalization. All singleton births in California June 22, 2020 – November 28, 2021. Source: California Department of Public Health.

Table A2. Linear probability fixed effects models predicting preterm birth. All singleton births in California June 2020-November 2021.

	Model 1<br>No controls	Model 2<br>WIth controls	Model 3<br>M2+COVID*race<br>interaction	Model 4<br>M2+COVID*education<br>interaction
COVID infection at time of delivery	0.028***	0.025***	0.025***	0.022***
	(0.003)	(0.003)	(0.004)	(0.007)
Education: Less than high school				
Education: High school graduate		-0.010***	-0.010***	-0.010***
		(0.002)	(0.002)	(0.002)
Education: Some college		-0.015***	-0.015***	-0.015***
		(0.002)	(0.002)	(0.002)
Education: BA		-0.032***	-0.032***	-0.032***
		(0.002)	(0.002)	(0.002)
Education: Graduate degree		-0.038***	-0.038***	-0.038***
		(0.003)	(0.003)	(0.003)
Race/ethnicity: Hispanic (omitted)				
Race/ethnicity: White		-0.012***	-0.012***	-0.012***
		(0.001)	(0.001)	(0.001)
Race/ethnicity: Black		0.021***	0.021***	0.021***
		(0.002)	(0.002)	(0.002)
Race/ethnicity: Asian		0.007***	0.007***	0.004â
		(0.002)	(0.002)	(0.002)
Race/ethnicity: Other		0.004â	0.004â	0.007***
		(0.002)	(0.002)	(0.002)
Foreign-born		-0.009***	-0.009***	-0.009***
		(0.001)	(0.001)	(0.001)
Age: 19 or younger				
Age: 20-24		-0.002	-0.002	-0.002
		(0.002)	(0.002)	(0.002)
Age: 25-29		0.005*	0.005*	0.005*
		(0.003)	(0.003)	(0.003)
Age: 30-34		0.016***	0.016***	0.016***
		(0.003)	(0.003)	(0.003)
Age: 35-39		0.030***	0.030***	0.030***
		(0.003)	(0.003)	(0.003)
Age: 40 and older		0.056***	0.056***	0.056***
		(0.003)	(0.003)	(0.003)
ZIP code disadvantage: Quartile 1				
ZIP code disadvantage: Quartile 2		0.008***	0.008***	0.008***
		(0.001)	(0.001)	(0.001)
ZIP code disadvantage: Quartile 3		0.012***	0.012***	0.012***
		(0.002)	(0.002)	(0.002)
ZIP code disadvantage: Quartile 4		0.016***	0.016***	0.016***
		(0.002)	(0.002)	(0.002)
Parity: First birth (reference)				
Parity: Second birth		-0.012***	-0.012***	-0.012***
		(0.001)	(0.001)	(0.001)
Parity: Third birth or higher		-0.001	-0.001	-0.001
		(0.001)	(0.001)	(0.001)
Covid infection*White			-0.002	
			(0.007)	
Covid infection*Black			0.005	
			(0.014)	
Covid infection*Asian			0.009	
			(0.011)	
Covid infection*Other race/ethnicity			-0.005	
			(0.014)	
Covid infection*HS graduate				-0.003
				(0.009)
Covid infection*Some college				0.007
				(0.008)
College infection*BA				0.004
				(0.010)
College infection*Graduate degree				0.002
				(0.013)
Constant	0.073***	0.088***	0.088***	0.088***
	(0.000)	(0.005)	(0.005)	(0.005)
Observations	544899	544899	544899	544899

Cluster robust standard errors in parentheses. + p<.10 * p<.05, ** p<.01, *** p<.001. COVID infection measured at time of labor and delivery hospitalization. All singleton births in California June 22, 2020 – November 28, 2021. Source: California Department of Public Health.

Table A3. Parameter estimates and significance testing, ARIMA models predicting changes in the preterm birth rate after the onset of the pandemic, all births and by educational attainment of the person giving birth. All singleton births in California January 1, 2014-November 28, 2021.

	Model 1 All, no covariates	Model 2 M1+covariates	Model 3 M2+delivery mode	Model 4 Excludes COVID infections	Model 5 Education: High school or less	Model 6 Education: Some college	Model 7 Education: BA or more
Mar16-Apr12 2020	-0.002	-0.001	-0.002	-0.002	0.001	-0.007â	-0.007â
	(0.002)	(0.002)	(0.002)	(0.002)	(0.003)	(0.004)	(0.004)
Apr13-May10	-0.005*	-0.005*	-0.004*	-0.004*	-0.009**	-0.000	-0.000
	(0.002)	(0.002)	(0.002)	(0.002)	(0.003)	(0.004)	(0.004)
May11-Jun7	-0.003	-0.004+	-0.004â	-0.003	-0.005	-0.008*	-0.008*
	(0.002)	(0.002)	(0.002)	(0.002)	(0.003)	(0.004)	(0.004)
Jun8-Jul5	0.003	0.002	0.002	0.003	0.004	0.001	0.001
	(0.002)	(0.002)	(0.002)	(0.002)	(0.003)	(0.004)	(0.004)
Jul6-Aug2	-0.004*	-0.005*	-0.005*	-0.006*	0.001	-0.012**	-0.012**
	(0.002)	(0.002)	(0.002)	(0.002)	(0.003)	(0.004)	(0.004)
Aug3-Aug30	0.000	-0.001	-0.001	0.000	-0.001	-0.002	-0.002
	(0.002)	(0.002)	(0.002)	(0.002)	(0.003)	(0.004)	(0.004)
Aug31-Sep27	-0.000	-0.002	-0.002	-0.001	-0.001	-0.002	-0.002
	(0.002)	(0.002)	(0.002)	(0.002)	(0.003)	(0.004)	(0.004)
Sep28-Oct25	-0.001	-0.003	-0.003	-0.002	0.002	-0.006	-0.006
	(0.002)	(0.002)	(0.002)	(0.002)	(0.003)	(0.004)	(0.004)
Oct26-Nov22	-0.007**	-0.008**	-0.008***	-0.008**	-0.006â	-0.008*	-0.008*
	(0.002)	(0.003)	(0.002)	(0.002)	(0.003)	(0.004)	(0.004)
Nov23-Dec20	-0.001	-0.003	-0.003	-0.002	-0.004	-0.001	-0.001
	(0.002)	(0.003)	(0.003)	(0.002)	(0.003)	(0.004)	(0.004)
Dec21 2020-Jan24 2021	0.004â	0.005â	0.005*	0.002	0.010**	0.003	0.003
	(0.002)	(0.003)	(0.003)	(0.002)	(0.003)	(0.004)	(0.004)
Jan25-Feb21	0.002	0.002	0.002	0.001	0.005	0.000	0.000
	(0.003)	(0.003)	(0.003)	(0.002)	(0.003)	(0.004)	(0.004)
Feb22-Mar21	0.000	0.000	0.001	0.000	0.003	-0.002	-0.002
	(0.003)	(0.003)	(0.003)	(0.003)	(0.003)	(0.004)	(0.004)
Mar22-Apr18	0.003	0.004	0.004	0.003	0.010*	-0.000	-0.000
	(0.004)	(0.004)	(0.004)	(0.004)	(0.005)	(0.006)	(0.006)
Apr19-May16	-0.001	-0.001	-0.001	-0.001	-0.004	-0.002	-0.002
	(0.004)	(0.004)	(0.004)	(0.004)	(0.005)	(0.006)	(0.006)
May17-Jun13	-0.004	-0.004	-0.004	-0.003	-0.003	-0.010	-0.010
	(0.004)	(0.004)	(0.004)	(0.004)	(0.005)	(0.007)	(0.007)
Jun14-Jul11	-0.004	-0.005	-0.004	-0.004	0.002	-0.004	-0.004
	(0.004)	(0.004)	(0.004)	(0.004)	(0.005)	(0.007)	(0.007)
Jul12-Aug8	-0.001	-0.002	-0.002	-0.002	0.001	-0.007	-0.007
	(0.004)	(0.004)	(0.004)	(0.004)	(0.005)	(0.007)	(0.007)
Aug9-Sep5 2021	0.003	0.002	0.003	0.001	0.004	-0.001	-0.001
	(0.004)	(0.004)	(0.004)	(0.004)	(0.005)	(0.007)	(0.007)
Sep6-Oct3	0.001	0.001	0.002	0.001	0.001	0.000	0.000
	(0.004)	(0.005)	(0.004)	(0.004)	(0.005)	(0.007)	(0.007)
Oct4-Oct31	-0.002	-0.003	-0.002	-0.003	0.000	-0.004	-0.004
	(0.004)	(0.005)	(0.004)	(0.004)	(0.005)	(0.007)	(0.007)
Nov1-Nov28	0.003	0.002	0.003	0.003	-0.001	0.006	0.006
	(0.004)	(0.005)	(0.005)	(0.004)	(0.005)	(0.008)	(0.008)
Age		-0.022	-0.016				
		(0.018)	(0.018)				
Age squared		0.000	0.000				
		(0.000)	(0.000)				
Race: White		-0.191**	-0.197***				
		(0.060)	(0.059)				
Race: Hispanic		-0.114*	-0.122*				
		(0.057)	(0.056)				
Race: Asian		-0.146*	-0.138*				
		(0.065)	(0.065)				
Race: Other		-0.267***	-0.251***				
		(0.073)	(0.072)				
Race: Black (ref.)							
BA+		0.013	0.016				
		(0.033)	(0.033)				
Foreign-born		-0.060*	-0.057*				
		(0.028)	(0.028)				
ZIP code disadvantage Q1		-0.015	-0.011				
		(0.043)	(0.042)				
ZIP code disadvantage Q2		0.037	0.030				
		(0.038)	(0.037)				
ZIP code disadvantage Q3		-0.016	-0.010				
		(0.031)	(0.030)				
ZIP code disadvantage Q4 (ref.)							
Cesarean section			0.075**				
			(0.024)				
Labor induction			0.023				
			(0.028)				

Cluster robust standard errors in parentheses. + p<.10 * p<.05, ** p<.01, *** p<.001. All singleton births in California January 1, 2014 – November 28, 2021. Preferred specification is multiplicative seasonal ARIMA model (0,1,1) with multiplicative seasonal component (0,1,0,52). COVID infection measured at time of delivery hospitalization.
Source: California Department of Public Health.

Table A4. Linear probability fixed effects models predicting COVID infection at the time of delivery and preterm birth restricting sample to birthing facilities with confirmed universal testing. Singleton births in California June 2020-November 2021.

	COVID infection	Preterm birth
COVID infection		0.027***
		(0.004)
Education: Less than high school (reference)		
Education: High school graduate	-0.007***	-0.010***
	(0.002)	(0.002)
Education: Some college	-0.006***	-0.014***
	(0.002)	(0.002)
Education: BA	-0.014***	-0.031***
	(0.002)	(0.003)
Education: Graduate degree	-0.017***	-0.041***
	(0.002)	(0.004)
Race: Hispanic (reference)		
Race: White	-0.013***	-0.013***
	(0.001)	(0.002)
Race: Black	-0.015***	0.018***
	(0.002)	(0.003)
Race: Asian	-0.019***	0.005**
	(0.002)	(0.002)
Race: Other	-0.011***	0.002
	(0.002)	(0.003)
Foreign-born	0.003**	-0.011***
	(0.001)	(0.001)
Age: 19 or younger		
Age: 20-24	0.002	-0.015***
	(0.003)	(0.004)
Age: 25-29	-0.005	-0.007
	(0.003)	(0.004)
Age: 30-34	-0.009*	0.004
	(0.003)	(0.004)
Age: 35-39	-0.012**	0.019***
	(0.004)	(0.005)
Age: 40 and older	-0.012***	0.041***
	(0.004)	(0.005)
ZIP code disadvantage: Quartile 1 (reference)		
ZIP code disadvantage: Quartile 2	0.005***	0.008***
	(0.001)	(0.002)
ZIP code disadvantage: Quartile 3	0.008***	0.011***
	(0.002)	(0.002)
ZIP code disadvantage: Quartile 4	0.011***	0.017***
	(0.002)	(0.002)
Parity: First birth (reference)		
Parity: Second birth	0.003***	-0.013***
	(0.001)	(0.001)
Parity: Third birth or higher	0.006***	-0.003â
	(0.001)	(0.001)
Constant	0.032***	0.099***
	(0.005)	(0.007)
Observations	313,212	304,104

Cluster robust standard errors in parentheses. + p<.10 * p<.05, ** p<.01, *** p<.001. COVID infection measured at time of labor and delivery hospitalization. All singleton births in California June 22, 2020 – November 28, 2021. Source: California Department of Public Health.

Table A5. Binary logistic fixed effects models predicting COVID infection at the time of delivery and preterm birth. All singleton births in California June 2020-November 2021.

VARIABLES	COVID infection	Preterm birth
COVID infection		0.317***
		(0.028)
Education: Less than high school (reference)		
Education: High school graduate	-0.123***	-0.125***
	(0.029)	(0.019)
Education: Some college	-0.147***	-0.186***
	(0.030)	(0.020)
Education: BA	-0.429***	-0.443***
	(0.037)	(0.023)
Education: Graduate degree	-0.751***	-0.544***
	(0.048)	(0.026)
Race: Hispanic (omitted)		
Race: White	-0.443***	-0.184***
	(0.026)	(0.016)
Race: Black	-0.432***	0.249***
	(0.043)	(0.022)
Race: Asian	-0.365***	0.116***
	(0.044)	(0.019)
Race: Other	-0.763***	0.060*
	(0.038)	(0.025)
Foreign-born	0.095***	-0.144***
	(0.021)	(0.013)
Age: 19 or younger	0.420***	-0.604***
	(0.070)	(0.044)
Age: 20-24	0.411***	-0.706***
	(0.044)	(0.025)
Age: 25-29	0.266***	-0.605***
	(0.040)	(0.020)
Age: 30-34	0.121**	-0.445***
	(0.039)	(0.019)
Age: 35-39	0.018	-0.256***
	(0.042)	(0.020)
Age: 40 and older (reference)		
ZIP code disadvantage: Quartile 1 (reference)		
	0.187***	0.121***
ZIP code disadvantage: Quartile 2	(0.030)	(0.017)
	0.287***	0.191***
ZIP code disadvantage: Quartile 3	(0.030)	(0.018)
	0.390***	0.243***
ZIP code disadvantage: Quartile 4	(0.033)	(0.020)
Parity: First birth (reference)		
Parity: Second birth	0.081***	-0.193***
	(0.022)	(0.013)
Parity: Third birth or higher	0.207***	-0.037**
	(0.024)	(0.014)
Constant	-2.389*	-2.070***
	(1.065)	(0.111)
Observations	551,049	543,077

Cluster robust standard errors in parentheses. + p<.10 * p<.05, ** p<.01, *** p<.001. COVID infection measured at time of labor and delivery hospitalization. All singleton births in California June 22, 2020 – November 28, 2021. Source: California Department of Public Health.

Table A6. Analyses predicting COVID infection and preterm birth including a larger set of covariates. Singleton births in California June 22, 2020 - November 28, 2021.

	1 With additional controls	2 + COVID*race interaction	3+COVID*education interaction	4 With controls + prenatal care
COVID infection	0.025***	0.023***	0.022***	0.020***
	(0.003)	(0.004)	(0.006)	(0.003)
Education: Less than high school (reference)				
Education: High school graduate	-0.010***	-0.010***	-0.010***	-0.006***
	(0.001)	(0.001)	(0.001)	(0.002)
Education: Some college	-0.014***	-0.014***	-0.015***	-0.007***
	(0.002)	(0.002)	(0.002)	(0.002)
Education: BA	-0.029***	-0.029***	-0.029***	-0.020***
	(0.002)	(0.002)	(0.002)	(0.002)
Education: Graduate degree	-0.035***	-0.035***	-0.035***	-0.026***
	(0.002)	(0.002)	(0.002)	(0.003)
Race: Hispanic (reference)				
Race: White	-0.012***	-0.012***	-0.012***	-0.011***
	(0.001)	(0.001)	(0.001)	(0.001)
Race: Black	0.017***	0.017***	0.017***	0.013***
	(0.002)	(0.002)	(0.002)	(0.002)
Race: Asian	0.006***	0.006***	0.007***	0.003*
	(0.002)	(0.002)	(0.002)	(0.002)
Race: Other	0.002	0.002	0.002	-0.001
	(0.002)	(0.002)	(0.002)	(0.002)
Foreign-born	-0.007***	-0.007***	-0.007***	-0.007***
	(0.001)	(0.001)	(0.001)	(0.001)
Age: 19 or younger				
Age: 20-24	-0.003	-0.003	-0.003	0.005*
	(0.002)	(0.002)	(0.002)	(0.002)
Age: 25-29	0.003	0.003	0.003	0.015***
	(0.003)	(0.003)	(0.003)	(0.003)
Age: 30-34	0.012***	0.012***	0.012***	0.025***
	(0.003)	(0.003)	(0.003)	(0.003)
Age: 35-39	0.025***	0.025***	0.025***	0.039***
	(0.003)	(0.003)	(0.003)	(0.003)
Age: 40 and older	0.048***	0.048***	0.048***	0.064***
	(0.003)	(0.003)	(0.003)	(0.004)
ZIP code disadvantage: Quartile 1 (reference)				
ZIP code disadvantage: Quartile 2	0.007***	0.007***	0.007***	0.006***
	(0.001)	(0.001)	(0.001)	(0.001)
ZIP code disadvantage: Quartile 3	0.011***	0.011***	0.011***	0.010***
	(0.002)	(0.002)	(0.002)	(0.001)
ZIP code disadvantage: Quartile 4	0.016***	0.016***	0.016***	0.014***
	(0.002)	(0.002)	(0.002)	(0.002)
Parity: First birth (reference)				
Parity: Second birth	-0.016***	-0.016***	-0.016***	-0.020***
	(0.001)	(0.001)	(0.001)	(0.001)
Parity: Third birth or higher	-0.009***	-0.009***	-0.009***	-0.016***
	(0.001)	(0.001)	(0.001)	(0.001)
Covid infection*White		0.005		
		(0.007)		
Covid infection*Black		0.007		
		(0.013)		
Covid infection*Asian		0.014		
		(0.009)		
Covid infection*Other race/ethnicity		0.002		
		(0.012)		
Covid infection*HS graduate			-0.001	
			(0.008)	
Covid infection*Some college			0.007	
			(0.008)	
College infection*BA			0.007	
			(0.009)	
College infection*Graduate degree			-0.002	
			(0.012)	
PGB smoked before pregnancy	0.045***	0.045***	0.045***	0.020***
	(0.004)	(0.004)	(0.004)	(0.004)
PGB pre-pregnancy diabetes	0.107***	0.107***	0.107***	0.121***
	(0.008)	(0.008)	(0.008)	(0.008)
PGB pre-pregnancy hypertension	0.094***	0.094***	0.094***	0.099***
	(0.006)	(0.006)	(0.006)	(0.006)
PGB asthma	0.009***	0.009***	0.009***	0.012***
	(0.003)	(0.003)	(0.003)	(0.003)
Previous preterm birth	0.191***	0.191***	0.191***	0.187***
	(0.008)	(0.008)	(0.008)	(0.007)
PGB large fibroid tumor	0.022***	0.022***	0.022***	0.024***
	(0.006)	(0.006)	(0.006)	(0.007)
Prenatal care began first trimester				0.043***
				(0.003)
Prenatal care visits 0-7 (reference category)				
Prenatal care visits 8-10				-0.115***
				(0.006)
Prenatal care visits 11-12				-0.164***
				(0.008)
Prenatal care visits 13-17				-0.174***
				(0.008)
Prenatal care visits 18+				-0.159***
				(0.008)
Constant	0.080***	0.080***	0.080***	0.159***
	(0.004)	(0.004)	(0.004)	(0.005)

Cluster robust standard errors in parentheses. + p<.10 * p<.05, ** p<.01, *** p<.001. COVID infection measured at time of labor and delivery hospitalization. All singleton births in California June 22, 2020 – November 28, 2021. Source: California Department of Public Health.

Table A7. Analyses predicting preterm birth adjusting for occupation-based proximity to others and occupation-based exposure to infection of both parents. Singleton births in California June 22, 2020 – November 28, 2021.

	COVID infection	Preterm birth
COVID infection during pregnancy		0.024***
		(0.003)
Education: Less than high school (reference)		
Education: High school graduate	-0.005***	-0.010***
	(0.001)	(0.002)
Education: Some college	-0.006***	-0.014***
	(0.001)	(0.002)
Education: BA	-0.011***	-0.030***
	(0.001)	(0.002)
Education: Graduate degree	-0.014***	-0.036***
	(0.002)	(0.003)
Race: Hispanic (omitted)		
Race: White	-0.012***	-0.011***
	(0.001)	(0.001)
Race: Black	-0.016***	0.020***
	(0.002)	(0.002)
Race: Asian	-0.018***	0.007***
	(0.002)	(0.002)
Race: Other	-0.012***	0.003
	(0.002)	(0.002)
Foreign-born	0.004***	-0.009***
	(0.001)	(0.001)
Age: Less than 19 years old (reference)		
Age: 19-23	0.001	-0.009*
	(0.003)	(0.004)
Age: 24-29	-0.004	-0.002
	(0.003)	(0.004)
Age: 30-34	-0.008**	0.010*
	(0.003)	(0.004)
Age: 35-38	-0.010**	0.023***
	(0.003)	(0.004)
Age: More than 38	-0.011***	0.043***
	(0.003)	(0.004)
ZIP code disadvantage: Quartile 1 (reference)		
ZIP code disadvantage: Quartile 2	0.004***	0.008***
	(0.001)	(0.001)
ZIP code disadvantage: Quartile 3	0.007***	0.012***
	(0.001)	(0.002)
ZIP code disadvantage: Quartile 4	0.010***	0.016***
	(0.001)	(0.002)
Parity: First birth (reference)		
Parity: Second birth	0.003***	-0.012***
	(0.001)	(0.001)
Parity: Third birth	0.006***	-0.002*
	(0.001)	(0.001)
Sixtile 1 parent 1's occupational proximity others (reference)		
Sixtile 2 parent 1's occupational proximity others	0.002*	-0.004**
	(0.001)	(0.002)
Sixtile 3 parent 1's occupational proximity others	0.001	0.000
	(0.001)	(0.002)
Sixtile 4 parent 1's occupational proximity others	0.003â[illegible]	0.002
	(0.001)	(0.002)
Sixtile 5 parent 1's occupational proximity others	0.002	-0.000
	(0.001)	(0.002)
Sixtile 6 parent 1's occupational proximity others	0.002	-0.008***
	(0.001)	(0.002)
Parent 1 proximity others unknown	-0.004	0.035***
	(0.003)	(0.004)

Parent 1 is homemaker	0.002	0.004*
	(0.001)	(0.002)
Parent 1 is student	0.001	-0.004
	(0.003)	(0.003)
Sixtile 1 parent 2's occupational proximity others (reference)		
Sixtile 2 parent 2's occupational proximity others	0.001	0.000
	(0.001)	(0.002)
Sixtile 3 parent 2's occupational proximity others	0.001	-0.001
	(0.001)	(0.002)
Sixtile 4 parent 2's occupational proximity others	0.001	0.001
	(0.001)	(0.002)
Sixtile 5 parent 2's occupational proximity others	0.002*	0.005**
	(0.001)	(0.002)
Sixtile 6 parent 2's occupational proximity others	0.002*	-0.002
	(0.001)	(0.002)
Parent 2 occupational proximity others unknown	0.058***	-0.208
	(0.007)	(0.157)
Parent 2 is homemaker	0.006**	0.004
	(0.002)	(0.003)
Parent 2 is student	0.002	-0.005
	(0.003)	(0.005)
Sixtile 1 parent 1's occupational exposure infection (reference)		
Sixtile 2 parent 1's occupational exposure infection	-0.000	-0.000
	(0.001)	(0.002)
Sixtile 3 parent 1's occupational exposure infection	-0.001	0.002
	(0.001)	(0.002)
Sixtile 4 parent 1's occupational exposure infection	0.001	0.007***
	(0.001)	(0.002)
Sixtile 5 parent 1's occupational exposure infection	0.000	0.007**
	(0.001)	(0.002)
Sixtile 6 parent 1's occupational exposure infection	0.004*	0.010***
	(0.002)	(0.002)
Parent 1 occupational exposure to infection unknown	0.000	-0.034***
	(0.003)	(0.004)
Sixtile 1 parent 2's occupational exposure infection (reference)		
Sixtile 2 parent 2's occupational exposure infection	0.002	-0.001
	(0.001)	(0.002)
Sixtile 3 parent 2's occupational exposure infection	-0.001	-0.001
	(0.001)	(0.001)
Sixtile 4 parent 2's occupational exposure infection	-0.000	0.001
	(0.001)	(0.002)
Sixtile 5 parent 2's occupational exposure infection	-0.000	-0.000
	(0.001)	(0.001)
Sixtile 6 parent 2's occupational exposure infection	0.001	0.001
	(0.001)	(0.002)
Parent 2's occupational exposure infection unknown	-0.045***	0.221
	(0.006)	(0.156)
Constant	0.020***	0.081***

Cluster robust standard errors in parentheses. + p<.10 * p<.05, ** p<.01, *** p<.001. COVID infection during pregnancy measured at time of labor and delivery hospitalization. All singleton births in California June 22, 2020 – November 28, 2021. Occupation information for both parents first recoded into detailed O*NET categories, then assigned 1-100 scores rating occupation-specific exposure to disease or infection and proximity to others included as O*NET work context descriptors of occupations (https://www.onetonline.org/find/descriptor/browse/Work_Context/). O*NET scores then recoded into sextiles. O*NET work context scores are not available for students and homemakers; additional indicator variables were added for these groups. Source: California Department of Public Health.

Figure A1. Time-series ARIMA analysis predicting changes in the preterm birth rate after the onset of the pandemic: Comparison of models dating births by date or birth and date of conception. All singleton births in California January 1, 2014-November 28, 2021.

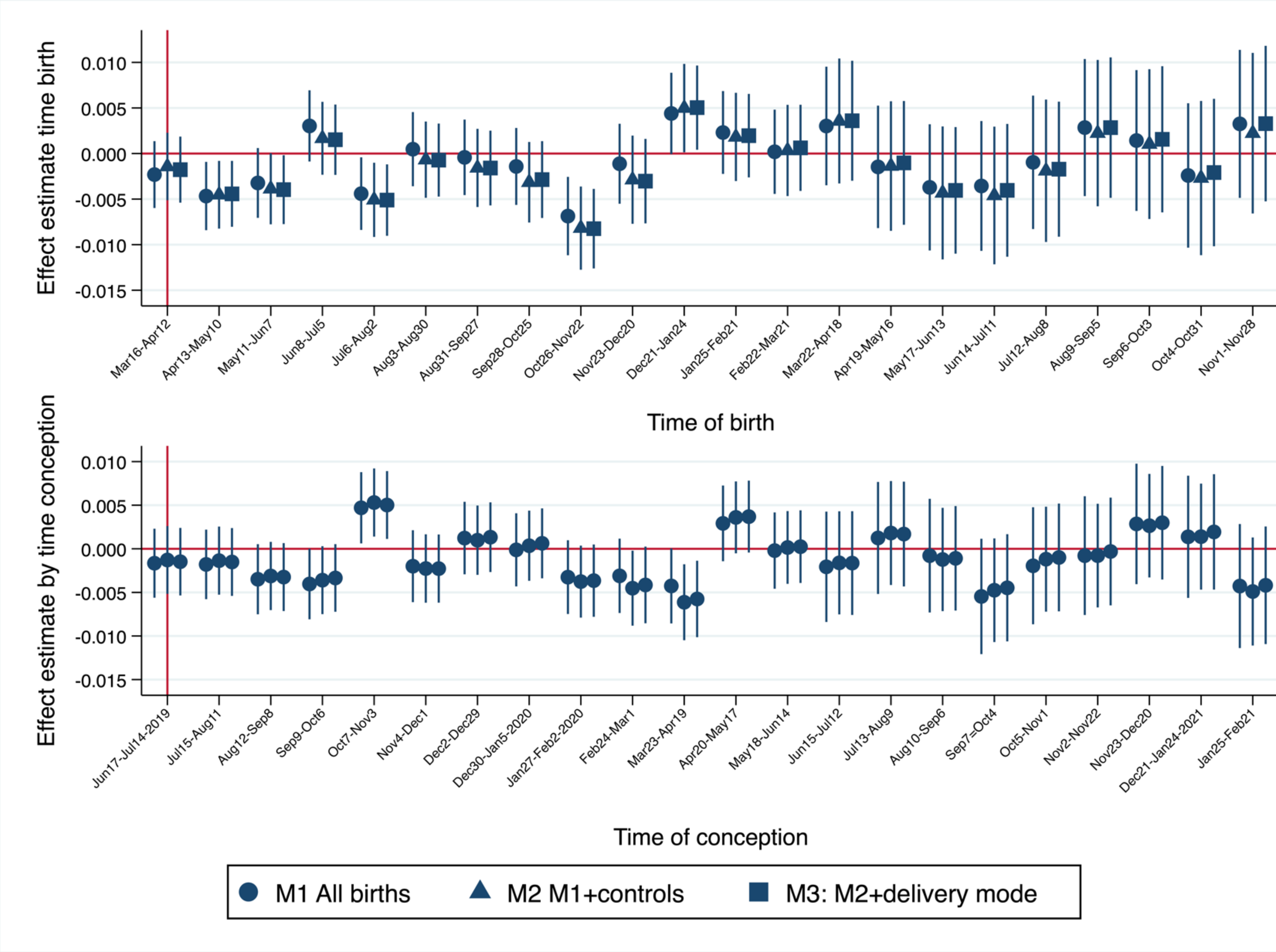

Solid markers are parameter estimates, vertical lines are 95 percent confidence intervals. Parameter estimates represent risk differences between observed rate of preterm birth and rate expected as function of temporal autocorrelation from January 2014 and February 2020 (pre-pandemic period). Preferred specification is multiplicative seasonal ARIMA model (0,1,1) with multiplicative seasonal component (0,1,0,52). Conceptions and births are temporally aligned assuming 39 weeks of gestation (the modal gestational age category in the sample). Conceptions provide a lagged indicator of trends given that preterm birth occurs by definition prior to modal gestational age.

Figure A2. Time-series analysis of preterm birth using Prais-Winsten regression to account for autocorrelation in time-series data. All singleton births in California January 1, 2014-November 28, 2021.

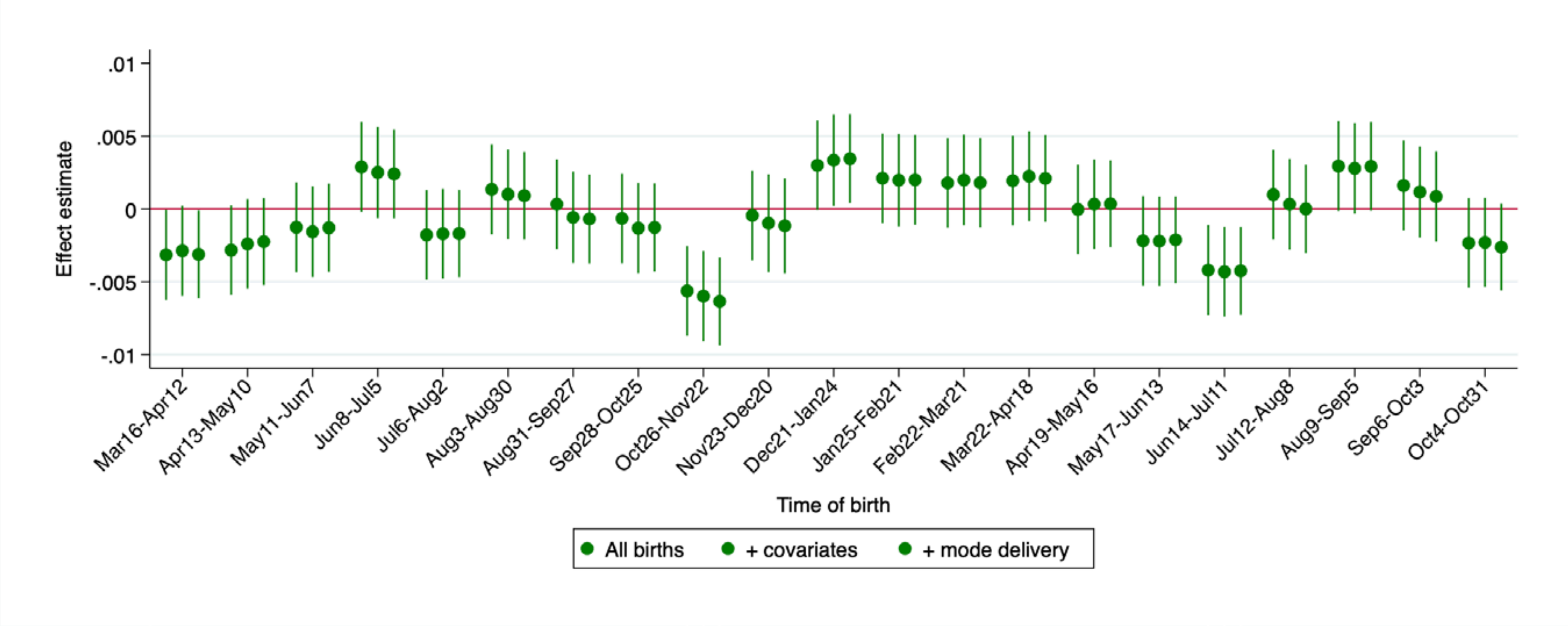

Solid markers are parameter estimates, vertical lines are 95 percent confidence intervals. Parameter estimates represent risk differences between observed rate of preterm birth and rate expected as function of temporal autocorrelation from January 2014 and February 2020 (pre-pandemic period). Model 2 includes the following covariates: mother's age, age squared, birth parity, education, race/ethnicity, foreign-born status, and ZIP code of residence SES quartile. Model 3 adds adjustment for cesarean delivery and induction of labor. Trend and seasonality extracted from time-series data by means of a Hodrick-Prescott filter with Lambda=270,400 and indicator variables for week of year, respectively.

Figure A3. Placebo time-series ARIMA analysis predicting preterm birth two calendar years earlier (March 2018-November 2019). All singleton births in California January 1, 2014-November 28, 2021.

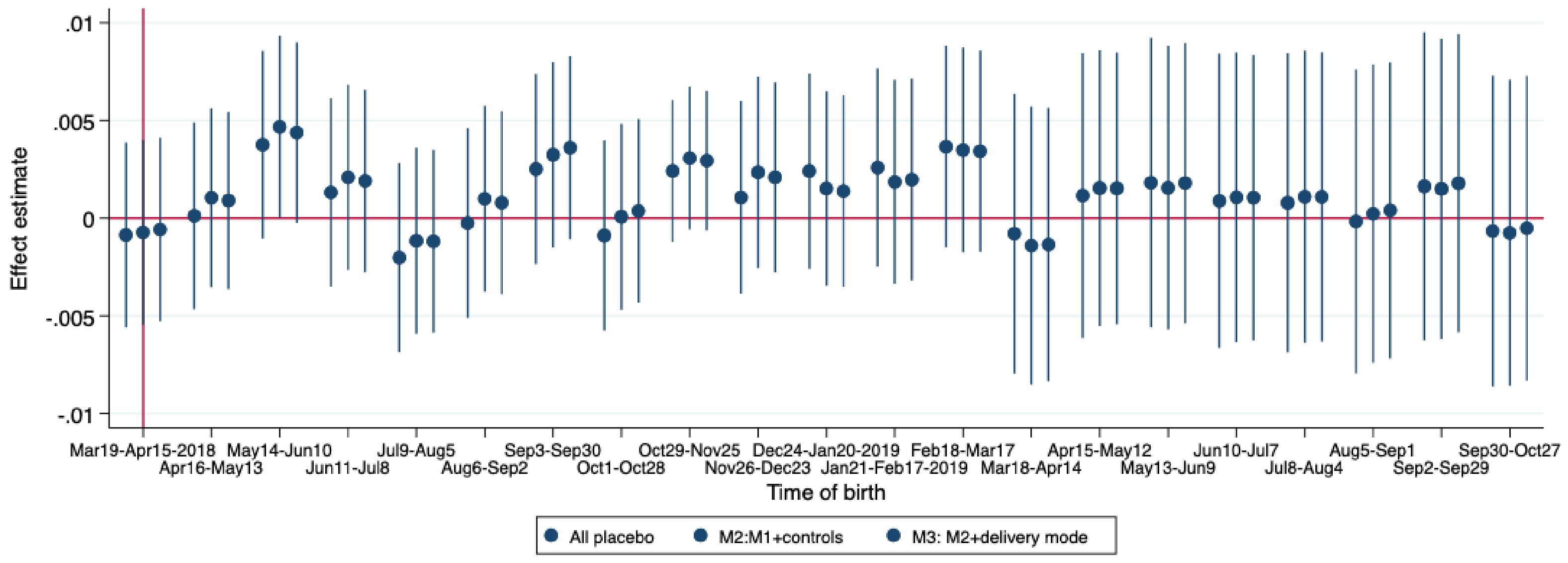

Solid markers are parameter estimates, vertical lines are 95 percent confidence intervals. Parameter estimates represent risk differences between observed rate of preterm birth and rate expected as function of temporal autocorrelation from January 2014 and February 2018. Model 2 includes the following covariates: mother's age, age squared, birth parity, education, race/ethnicity, foreign-born status, and ZIP code of residence SES quartile. Model 3 adds adjustment for cesarean delivery and induction of labor.
